# CircRFWD3 promotes HNSCC metastasis by modulating miR-27a/b/PPARγ signaling

**DOI:** 10.1038/s41420-022-01066-6

**Published:** 2022-06-11

**Authors:** Zihao Wei, Ying Wang, Jiakuan Peng, Honglin Li, Junjie Gu, Ning Ji, Taiwei Li, Xikun Zhou, Xin Zeng, Jing Li, Qianming Chen

**Affiliations:** 1 State Key Laboratory of Oral Diseases, National Clinical Research Center for Oral Diseases, Chinese Academy of Medical Sciences Research Unit of Oral Carcinogenesis and Management, West China Hospital of Stomatology, Sichuan University, Chengdu, Sichuan 610041 P.R. China; 2 State Key Laboratory of Biotherapy and Cancer Center, West China Hospital, Sichuan University and Collaborative Innovation Center for Biotherapy, Chengdu, 610041 China

**Keywords:** Oral cancer, Oral cancer

## Abstract

Head and neck squamous cell carcinoma (HNSCC) is the sixth most common cancer in the world, the 5-year survival rate of patients with HNSCC is still about 50% due to frequent metastasis and recurrence. Circular RNAs (circRNAs) have been characterized as key regulators of gene expression in numerous malignancies. However, the role of circRNA in HNSCC metastasis remains largely unknown. Here, we demonstrated that the circRFWD3 was significantly upregulated in HNSCC tissues and cell lines by circRNA microarray analysis and qPCR. Notably, high expression of circRFWD3 is related to highly aggressive HNSCC cell lines and lymph node metastasis in HNSCC patients. After that, Sanger sequencing, RNase R, and actinomycin D assay were performed to verify the ring structure of circRFWD3. Then functional experiments found it could promote the metastasis of HNSCC cells both in vitro and in vivo. Mechanistically, a dual-luciferase reporter assay, FISH, RIP, RNA pull-down, RNA-seq, and western blot experiments were employed and found that circRFWD3 served as a miRNAs sponge for miR-27a/27b, leading to the upregulation of PPARγ, and then promoted HNSCC metastasis via NF-κB/MMP13 pathway. Finally, ISH and IHC were carried out to determine the expression levels and clinical significances of circRFWD3 and PPARγ in clinical cohorts of HNSCC. According to the analysis results from two independent HNSCC cohorts, upregulated expression of circRFWD3 and PPARγ were positively associated with worse survival in patients with HNSCC. Overall, our results uncover that circRFWD3 acts a critical role in promoting the aggressiveness of HNSCC cells and is a prognostic marker for the disease, indicating that circRFWD3 may act as a potential therapeutic target in HNSCC.

## Introduction

Head and neck squamous cell carcinoma (HNSCC), a kind of malignant tumor originating from the oral, pharynx, throat, and sinus mucosal epithelium [1], is the sixth most common cancer in the world [2], accounting for 4–7% of all malignancies [3, 4], with 890,000 deaths and even over 450,000 new cases in 2018 worldwide [5]. Despite advances in treatment, the 5-year overall survival (OS) rate of patients with HNSCC remains ~50% due to its frequent metastasis and recurrence, strong aggressiveness, and difficulty in early diagnosis [6–9]. Therefore, understanding the specific mechanisms of progression and metastasis and discovering the molecular predictive targets for metastasis and prognosis of HNSCC is a significant strategy for HNSCC intervention.

Circular RNAs (circRNAs) have recently been defined as endogenous noncoding RNAs that originate from back-splicing of precursor mRNAs and are characterized by single-stranded and closed covalent loops without 5′-3′ polarity. Compared with their linear RNA counterparts, circRNAs are more resistant to exonucleases and more stable [10]. CircRNAs show important biological functions mainly by acting as microRNA sponges, regulating protein function or gene transcription, splicing, and translation [11]. Numerous studies have indicated that the circRNA/miRNA/mRNA signaling axis exerts multiple functions in many physiologic and pathologic conditions, especially in cancers [12]. Recently, several circRNAs have been intensively characterized in HNSCC [13–15], but a great deal of circRNAs have not been explored, and the exact mechanism of circRNAs in tumor metastasis remains to be further explored.

In this study, with the application of circRNA microarray, we identified a novel unique RFWD3-derived circRNA, hsa_circ_101877 (termed circRFWD3), which showed significant upregulation in HNSCC. We found that circRFWD3 was a proto-oncogenic circRNA that promoted the migration and invasion ability of HNSCC. In particular, circRFWD3 served as a miRNA sponge for miR-27a/b, resulting in upregulation of PPARγ and then promoting metastasis of HNSCC via the NF-κB/MMP13 pathway. CircRFWD3 and PPARγ were positively associated with metastasis and could predict a worse prognosis in patients with HNSCC. These findings indicated that circRFWD3 might function as a promising therapeutic marker for HNSCC.

## Results

### CircRFWD3 is overexpressed in HNSCC tissues and cell lines

**Fig. 1:**
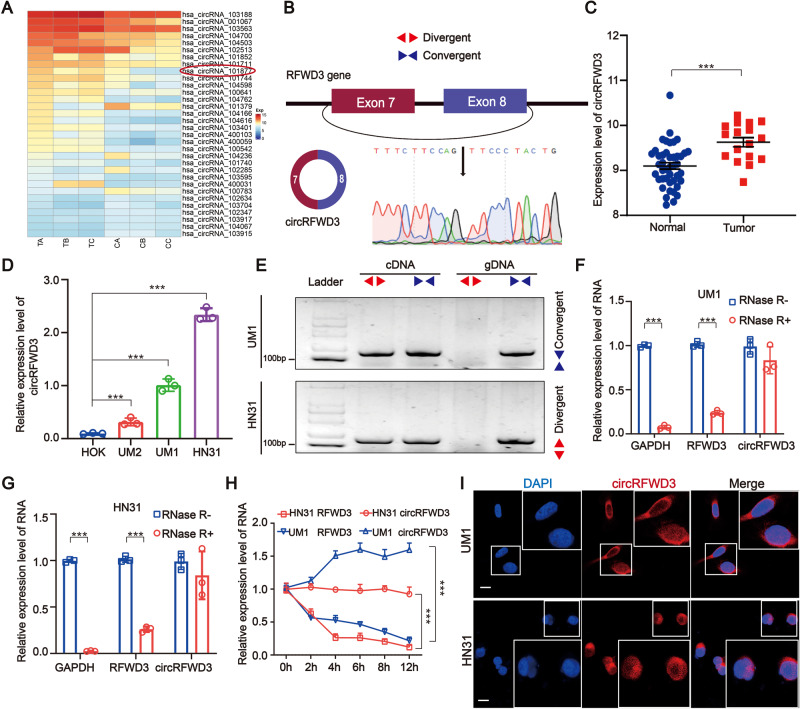
Screen and characterization of circRFWD3 in HNSCC. **A** Clustered heatmap showing the 32 differentially expressed circRNAs in three groups of human HNSCC tissues and adjacent normal tissues (red indicates upregulated circRNAs, and blue indicates downregulated circRNAs). The rows represent groups of HNSCC tissue samples (C, normal tissue; T, tumor tissue), and columns represent circRNAs’ numerical ID obtained from circBase. **B** The genomic loci of the RFWD3 gene and circRFWD3 were primarily transcribed from exons 7 and 8 of RFWD3, which were detected by qRT-PCR and validated by Sanger sequencing amplified with divergent primers. **C**, **D** The expression level of circRFWD3 in 43 normal tissues and 17 HNSCC tissues (data from TCGA) and in normal HOK cells and three HNSCC cell lines. **E** Agarose gel electrophoresis with RT-PCR products using divergent and convergent primers in both gDNA and cDNA. Red arrows showed that divergent primers could amplify circRFWD3 from cDNA. **F**, **G** qRT-PCR was used to determine the abundance of circRFWD3 and linear RFWD3 mRNA in UM1 and HN31 cells treated with RNase R. Results demonstrated circRFWD3 was tolerant to RNase R. **H** CirRFWD3 resistance to actinomycin D (ActD) was detected by the qRT-PCR assay. **I** RNA FISH showed that circRFWD3 located in the cytoplasm in UM1 and HN31 cells, scale bars, 10 μm. Data were shown as the mean value ± SD of three independent experiments. The asterisks indicate significant differences (Student’s *t*-tests, *P < 0.05, **P < 0.01, ***P < 0.001).

Nine pairs of HNSCC tissues were divided into groups of three and paired adjacent nontumor tissues were also divided into corresponding groups of three. Then a circRNA microarray was performed to identity significantly expressed circRNAs in HNSCC. After screening, we obtained 32 circRNAs, including 23 upregulated and nine downregulated circRNAs (Fig. 1A). Among those significantly upregulated circRNAs, circRFWD3 (also called hsa_circ_101877, or hsa_circ_0004519) was upregulated 4.7 times in HNSCC. Therefore, we chose circRFWD3 as the object of our study. CircRFWD3 is transcribed from the gene RFWD3 on chromosome 16, resulting from back-splicing of exons 7 and 8 with a spliced sequence length of 347 bp. Sanger sequencing further confirmed the back-splicing junction site of circRFWD3 (Fig. 1B). In addition, PCR analysis confirmed that divergent primers could amplify circRFWD3 from cDNA, but not gDNA (Fig. 1E). Furthermore, compared with normal tissues and cell lines, qRT-PCR assays illustrated prominently higher expression of circRFWD3 in HNSCC tissues and cell lines, which verified the upregulation of circRFWD3 expression in HNSCC (Fig. 1C, D). In particular, the higher expression of circRFWD3 was steadily shown in UM1 and HN31 cells (*P* < 0.0001), which were then chosen to perform the following experiments. RNase R digestion resistance demonstrated the circular property of circRFWD3 (Fig. 1F, G). Actinomycin D treatment confirmed that the half-life of circRFWD3 was longer than that of RFWD3 mRNA in both UM1 and HN31 cells (Fig. 1H). Furthermore, the subcellular localization of circRFWD3 in UM1 and HN31 cells was analysed via RNA FISH assay, demonstrating that the circRFWD3 is principally localized in the cytoplasm (Fig. 1I). In conclusion, these results strongly indicated that circRFWD3 was remarkably upregulated in HNSCC, mainly localized in the cytoplasm.

### CircRFWD3 promotes the migration and invasion ability of HNSCC cells both in vitro and vivo

**Fig. 2:**
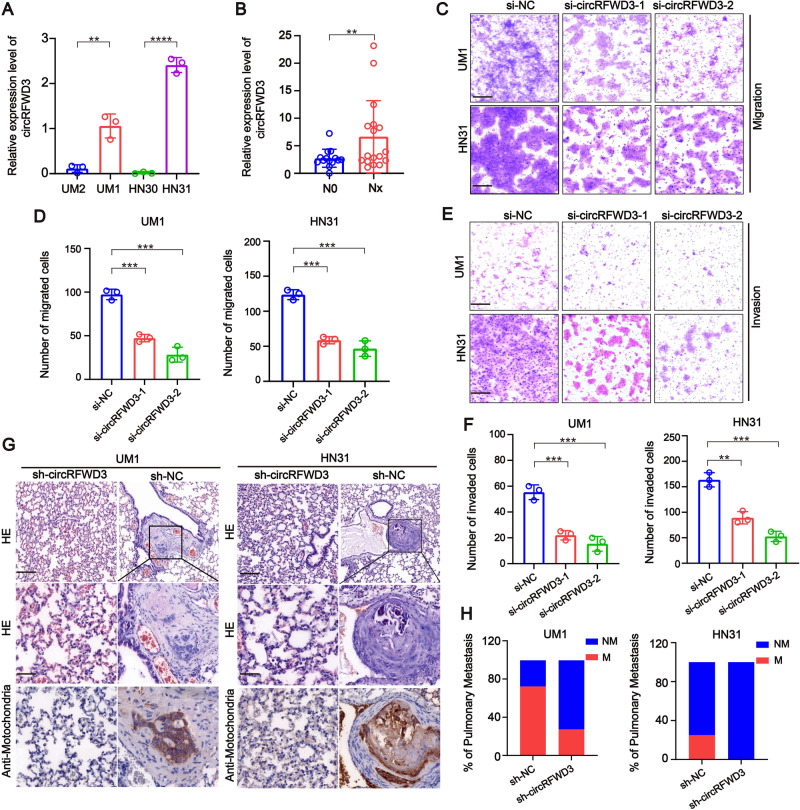
CircRFWD3 could enhance the migration and invasion ability of HNSCC cells in vitro and in vivo. **A** qRT-PCR assay showed the expression level of circRFWD3 in different HNSCC cell lines (UM1, UM2, HN30, and HN31). **B** A clinical cohort of nonmetastatic and metastatic HNSCC patient samples indicated significantly high expression of circRFWD3 in metastatic HNSCC patients (N0 = no lymph node metastasis, *n* = 15; Nx lymph node metastasis, *n* = 15). **C**, **D** The migration ability of UM1 and HN31 cells with silenced circRFWD3 was examined by transwell assays, scale bar, 100 μm. **E**, **F** The invasion ability of UM1 and HN31 cells by silencing circRFWD3 was examined by transwell assays, scale bar, 100 μm. **G** H&E and IHC analysis determined the metastatic tumor nodules in the lungs of mice injected with sh-NC or sh-circRFWD3 stably transfected UM1 and HN31 cells, scale bar, upper 100 μm, middle 10 μm. **H** Statistical graphs showed that the metastatic rate of the UM1 sh-NC group was 72.3% (*n* = 11), UM1 sh-cirCRFWD3 group was 27.3% (*n* = 11), HN31 sh-NC group was 25% (*n* = 12), and HN31 sh-cirCRFWD3 group was 0% (*n* = 12). NM no pulmonary metastasis, M pulmonary metastasis; Scale bar, 100 μm. Data were shown as the mean value ± SD of three independent experiments. The asterisks indicate significant differences (Student’s *t*-tests, *P < 0.05, **P < 0.01, ***P < 0.001).

We noticed that the expression of circRFWD3 in high-metastatic UM1 cells was higher than that in low-metastatic UM2 cells, suggesting that circRFWD3 may be involved in tumor metastasis and promote tumor progression. As expected, circRFWD3 was also more abundant in metastasis-derived HN31 cells than in matched primary tumor-derived HN30 cells (Fig. 2A). In addition, we observed that the expression level of circRFWD3 was observably higher in HNSCC tissues extracted from patients with lymph node metastasis than in nonmetastatic HNSCC tissues (Fig. 2B). Then, two siRNAs were designed to silence circRFWD3 without influencing RFWD3 mRNA levels in HNSCC cells (Appendix Fig. 1A, B), si-circRFWD3-2 was chosen for the following intravital investigations on account of its high inhibitory efficiency. The results of transwell assays showed that the migration and invasion abilities of UM1 and HN31 cells were remarkably restricted by the downregulation of circRFWD3 (Fig. 2C–F).

Next, to verify the circRFWD3 dependent on HNSCC cell metastasis in vivo, we adopted a lung metastasis mouse model. Anti-mitochondria, which is an excellent marker for human cells in xenograft model research, was used to detect metastasis in mouse lung tissues. The lungs of mice transplanted with sh-circRFWD3 rarely harbored metastatic tumor nodules, while the lungs of mice transplanted with sh-NC cells were widely occupied by visible metastatic tumors (Fig. 2G, H). These data distinctly showed that the decreased expression of circRFWD3 could visibly weaken the metastasis of HNSCC.

### CircRFWD3 promotes the migration and invasion ability of HNSCC cells by sponging both miR-27a and miR-27b

**Fig. 3:**
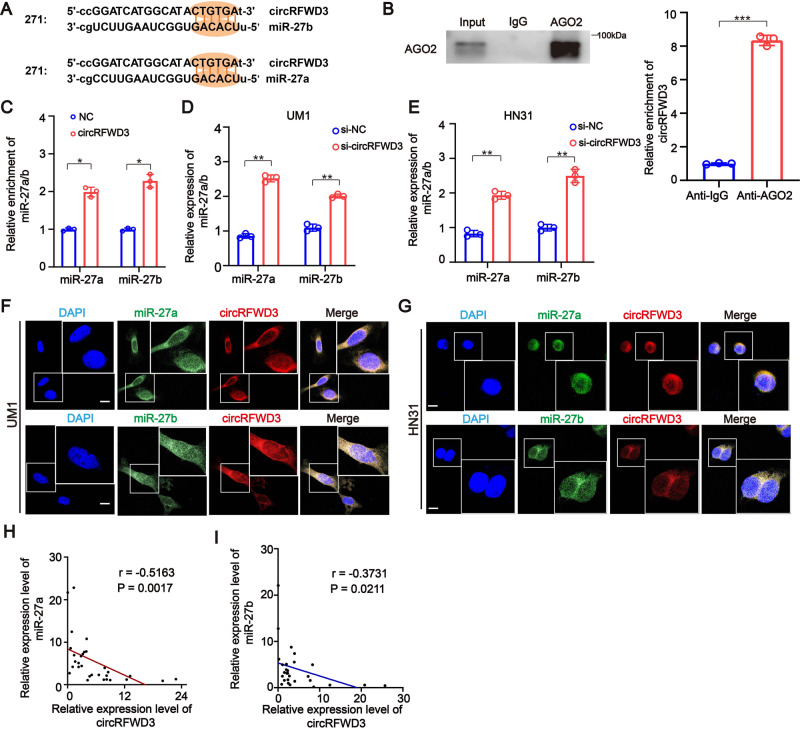
Both miR-27a and miR-27b could directly bound to circRFWD3. **A** Schematic illustration showing binding site sequences between circRFWD3 and miR-27a/b. **B** RIP assay showed that circRFWD3 was pulled down by AGO2 with a much greater enrichment in UM1 cells. The expression levels of circRFWD3 in RIP products were assessed by qRT-PCR. **C** RNA pull-down assay revealed exhibited the direct binding of circRFWD3 and miR-27a/b in UM1 cells. The expression levels of miR-27a and miR-27b in RNA pull-down products were assessed by qRT-PCR. Data were normalized to the control probe. **D**, **E** qRT-PCR assays showed that inhibition of circRFWD3 expression could significantly increase the expression of miR-27a and miR-27b in UM1 and HN31 cells. **F**, **G** RNA FISH showed colocalization of circRFWD3 and miR-27a/b in UM1 and HN31 cells, scale bar, 10 μm. **H**, **I** Pearson correlation analysis indicated that the expression of circRFWD3 was negatively correlated with miR-27a/b in patients with HNSCC (*n* = 15 and 15, respectively). Data were shown as the mean value ± SD of three independent experiments. The asterisks indicate significant differences (Student’s *t*-tests, *P < 0.05, **P < 0.01, ***P < 0.001).

To address whether circRFWD3 might serve as a sponge for miRNAs in HNSCC, we analyzed and predicted the miRNAs that circRFWD3 could bind to using two publicly available predictive tools, miRanda [16] and TargetScan [17]. According to the analysis, hsa-miR-27a-3p (miR-27a) and hsa-miR-27b-3p (miR-27b) were downstream targets of circRFWD3, which was consistent with the positions of supposed binding sites within the circRFWD3 sequence (Fig. 3A). Then, RIP assays indicated that circRFWD3 was availably pulled down by AGO2 protein in HNSCC cells (Fig. 3B), which indicated that circRFWD3 could act as a miRNA sponge [18]. To further confirm the interaction between circRFWD3 and miR-27a/b, we designed a biotinylated probe that can bind to the sequence of circRFWD3 which contains the back-splicing site. The RNA pulldown results showed that the biotin-labeled circRFWD3 probe could capture more miR-27a/b than the biotin-labeled negative control probe (Fig. 3C). Furthermore, the qRT-PCR assay showed that inhibition of circRFWD3 expression could significantly increase the expression of miR-27a/b (Fig. 3D, E). Additionally, RNA FISH assays demonstrated that circRFWD3 and miR-27a/b were colocalized in the cytoplasm (Fig. 3F, G). In addition, we found a negative correlation between circRFWD3 and miR-27a/b in our clinical cohort tissues (Fig. 3H, I). These results indicated that circRFWD3 could directly interact with and negatively modulate miR-27a/b in HNSCC, suggesting that circRFWD3 may regulate the migration and invasion ability of HNSCC through miR-27a/b.

**Fig. 4:**
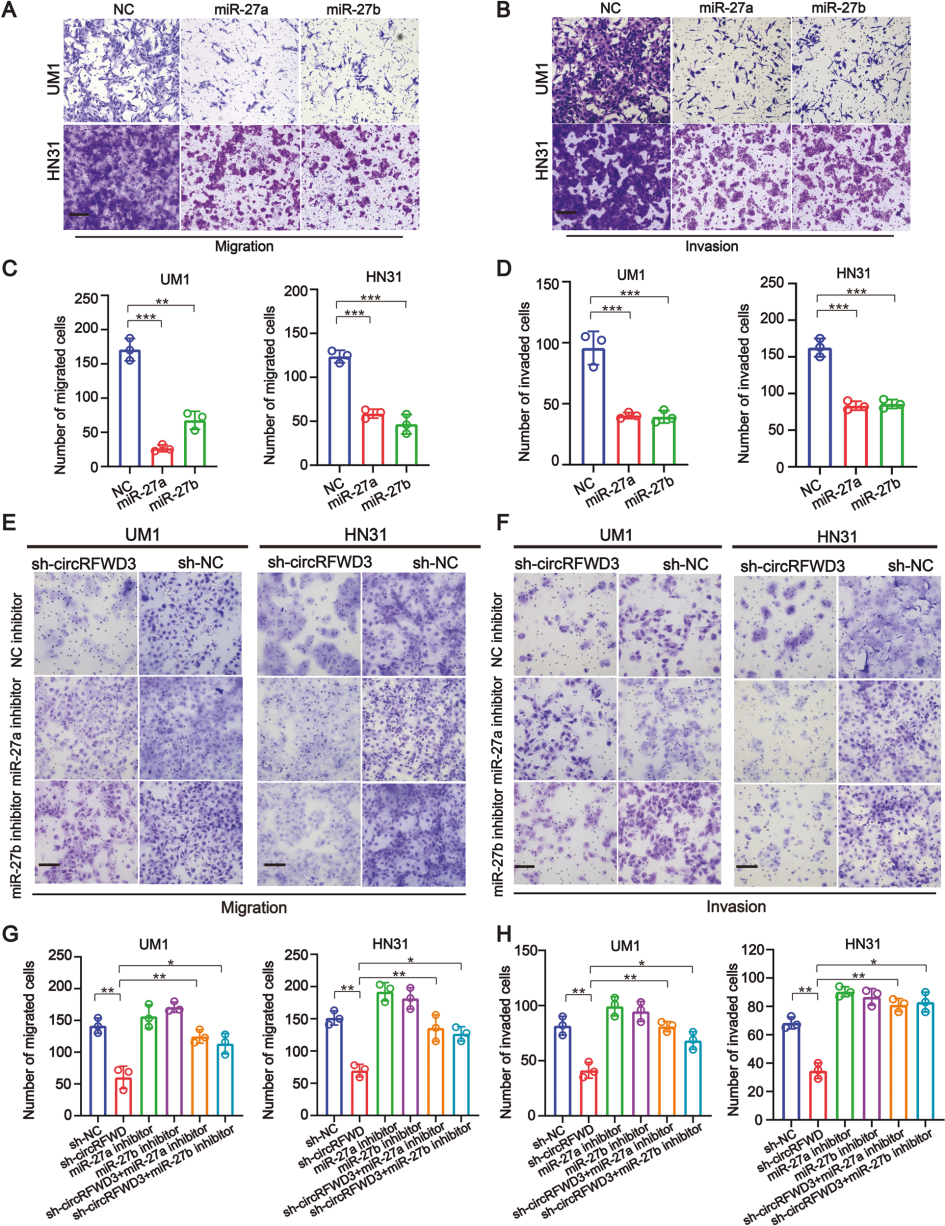
Both miR-27a and miR-27b could reverse the tumor metastasizing-effects of circRFWD3 in HNSCC cells. **A**, **C** Transwell assays indicated that the migration ability of UM1 and HN31 cell were observably restricted by transfection of miR-27a and miR-27b mimics. **B**, **D** Transwell assays indicated that the invasive capabilities of UM1 and HN31 cell were observably restricted by transfection of miR-27a and miR-27b mimics. **E**–**H** Transwell assays revealed that miR-27a and miR-27b inhibitors could rescue the inhibitory effects of circRFWD3 knockdown of migration and invasion in UM1 and HN31 cells. Scale bar, 100 μm. data were shown as the mean value ± SD of three independent experiments. The asterisks indicate significant differences (Student’s *t*-tests, *P < 0.05, **P < 0.01, ***P < 0.001).

To further investigate the exact role of miR-27a/b and their functional interaction with circRFWD3, miR-27a/b mimics or miR-27a/b inhibitors were designed, and qRT-PCR assays showed their higher enhancement or knockdown efficiency (Appendix Fig. 2A–D). Transwell assays indicated that the migration and invasion ability of HNSCC cells was observably restricted by transfection of miR-27a/b mimics (Fig. 4A–D). Consistently, miR-27a or miR-27b inhibitors could counteract the inhibitory roles of circRFWD3 knockdown in the migration and invasion ability of HNSCC cells (Fig. 4E–H). Collectively, these experiments demonstrated that circRFWD3 could serve as a ceRNA binding miR-27a/b to promote the migration and invasion ability of HNSCC.

### PPARγ is a downstream target of both miR-27a and miR-27b in HNSCC

**Fig. 5:**
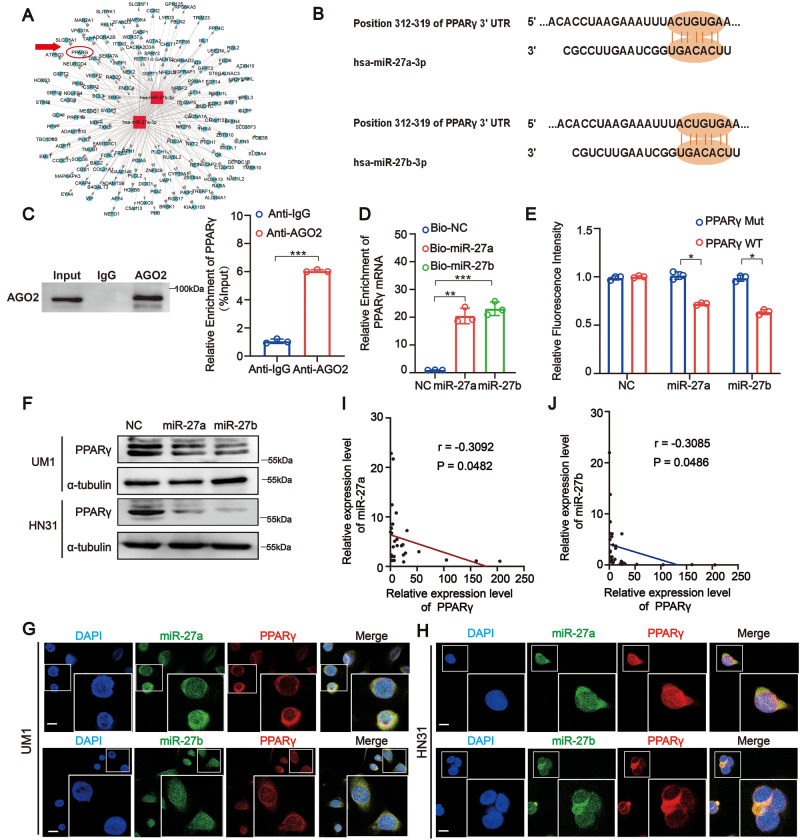
PPARγ is a direct target of both miR-27a and miR-27b in HNSCC. **A** The network of target genes of miR-27a and miR-27b, the red circle represents PPARγ. **B** Schematic illustration showed binding site sequences between PPARγ and miR-27a/27b. **C** RIP assay showed PPARγ mRNA was effectively pulled down by AGO2 with a much greater enrichment in UM1 cells. **D** RNA pull-down assay exhibited for the direct bonding of PPARγ and miR-27a/27b in UM1 cells. **E** Dual-luciferase assays were conducted using PPARγ wild type (WT) and mutant (MUT) 3′UTR reporter constructs to show that the human PPARγ 3′UTR region contains miR-27a and miR-27b binding sites. (Data were normalized to that with the control probe, showing the mean ± SD of three experiments). **F** Western Blot assay showed miR-27a and miR-27b mimic could significantly reduce expression of PPARγ in UM1 and HN31 cells at the level of proteins. **G**, **H** RNA FISH showed colocalization of PPARγ and miR-27a/27b in UM1 and HN31 cells (Scale bar, 10 μm). **I**, **J** Pearson correlation analysis indicated miR-27a and miR-27b expression was negatively correlated with PPARγ expression in patients with HNSCC (*n* = 15 and 15, respectively). Data were shown as the mean value ± SD of three independent experiments. The asterisks indicate significant differences (Student’s *t*-tests, *P < 0.05, **P < 0.01, ***P < 0.001).

To identify the potential targets of miR-27a/b in HNSCC, three algorithms: TargetScan [19], miRbase [20], and miRanda [16] were utilized. Based on the predicted results, we obtained a putative network of target genes of miR-27a/b (Fig. 5A). The PPARγ 3′UTR contains the reverse complementary base sequence of miR-27a/b (Fig. 5B). We speculated that PPARγ is a target of both miR-27a and miR-27b in HNSCC. We then verified this hypothesis through a series of experiments. First, RIP assays pointed out that PPARγ mRNA was effectively enriched by the AGO2 antibody in HNSCC cells (Fig. 5C). Afterward, the results of the RNA pulldown assay showed that the biotin-labeled miR-27a and miR-27b probes could capture more PPARγ mRNA than the biotin-labeled negative control probe (Fig. 5D). In addition, the results of the luciferase reporter assay revealed that transfection of miR-27a or miR-27b mimics dramatically reduced the luciferase activity of the WT PPARγ 3′UTR sequence but not that of the mutant PPARγ 3′UTR sequence in HNSCC cells (Fig. 5E). Moreover, we found that compared with the miR-NC group, miR-27a/b overexpression resulted in a remarkable downregulation of both mRNA and protein levels of PPARγ in HNSCC cells (Fig. 5F and Appendix Fig. 3A, B). We also found that miR-27a/b and PPARγ colocalized in the cytoplasm (Fig. 5G, H). In addition, clinical cohort analysis showed a negative correlation between miR-27a/b and PPARγ (Fig. 5I, J). These results indicated that both miR-27a and miR-27b could directly target and reduce the expression of PPARγ.

### CircRFWD3 augmented PPARγ/NF-κB/MMP13 signaling axis via sponging miR-27a/b in HNSCC

**Fig. 6:**
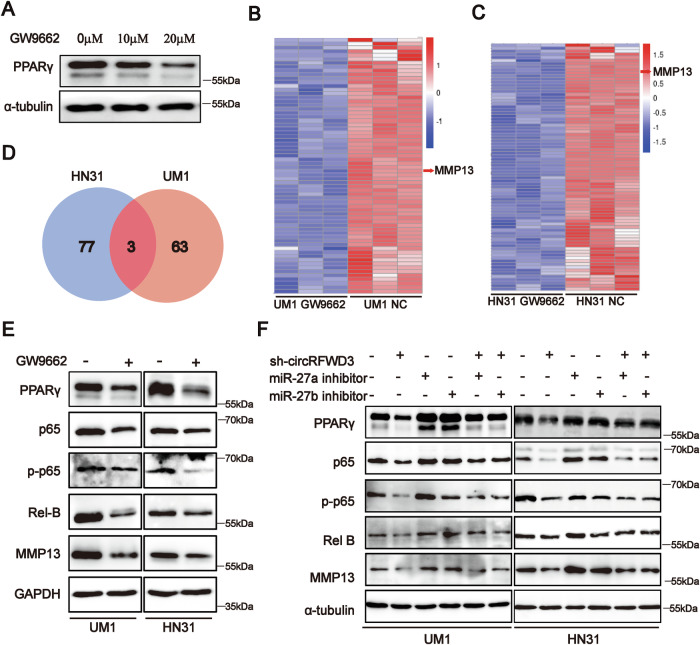
CircRFWD3 regulated PPARγ/NF-κB/MMP13 signal pathway via miR-27a/27b in HNSCC. **A** Western blot showed that PPARγ expression was decreased after GW9662 treatment in HN31 cells. **B**, **C** Clustered heatmap of a significant differentially expressed gene in UM1 and HN31 cells treated by GW9662. Each sample contained a mixture of three repeats. **D** Venn diagram showed MMP13, STRA6, and PTPRH were downregulated after GW9662 treatment in both HN31 and UM1 cells. **E** Western blot analysis showed inhibition of PPARγ could decrease the expression level of p65, p-P65, Rel B, and MMP13. **F** Rescue analysis indicated that miR-27a and miR-27b inhibitors could alleviate the reduction of PPARγ, p65, p-P65, Rel B, and MMP13 by inhibiting the expression of circRFWD3. Data were shown as representative of one experiment with three independent biological replicates.

Subsequently, we treated HNSCC cells with the PPARγ inhibitor, GW9662 [21], and the western blot results showed that the expression of PPARγ was decreased (Fig. 6A). Transcriptome analysis was then performed to identify the downstream targets mediated by PPARγ. A total of 20 mRNAs were upregulated and 66 mRNAs were downregulated in PPARγ-repressed UM1 cells, while 57 mRNAs were upregulated and 80 mRNAs were downregulated in PPARγ-repressed HN31 cells (Fig. 6B, C and Appendix Tables S5, 6). Considering that PPARγ should be positively correlated with HNSCC metastasis based on our existing data, we, therefore, focused on the downregulated genes. Among these differentially downregulated mRNAs, MMP13, STRA6, and PTPRH were downregulated in both HN31 and UM1 cells (Fig. 6D). Among them, MMP13 was most remarkably downregulated, belongs to the matrix metalloproteinase family, and contributes to tumor metastasis [22]. Furthermore, KEGG pathway enrichment analyses showed their significant enrichment in the IL-7 signaling pathway, which is involved in tumor migration and invasion, such as the NF-κB pathway and MAPK pathway in HNSCC cell lines (Appendix Fig. 3C, D). Western blot analysis indicated that inhibition of PPARγ decreased the expression levels of p65, p-p65, Rel B, and MMP13 (Fig. 6E and Appendix Fig. 3F), as well as MMP2 and MMP7 in UM1 and HN31 cells (Appendix Fig. 3E). The above results indicated that PPARγ could regulate the NF-κB/MMP13 pathway in HNSCC.

Additionally, we found that the protein levels of PPARγ were remarkably decreased in sh-circRFWD3-transfected HNSCC cells, and their expression could be restored by miR-27a or miR-27b inhibitors. Similarly, the protein levels of p65, p-p65, Rel B, and MMP13 were also significantly decreased in sh-circRFWD3-transfected UM1 and HN31 cells, and miR-27a and miR-27b inhibitors abolished their downregulation induced by sh-circRFWD3 (Fig. 6F). These findings demonstrated that circRFWD3 regulated the PPARγ/NF-κB/MMP13 signaling pathway via miR-27a/b in HNSCC.

### CircRFWD3 and PPARγ were positively associated with metastasis and could predict a worse prognosis in patients with HNSCC

**Fig. 7:**
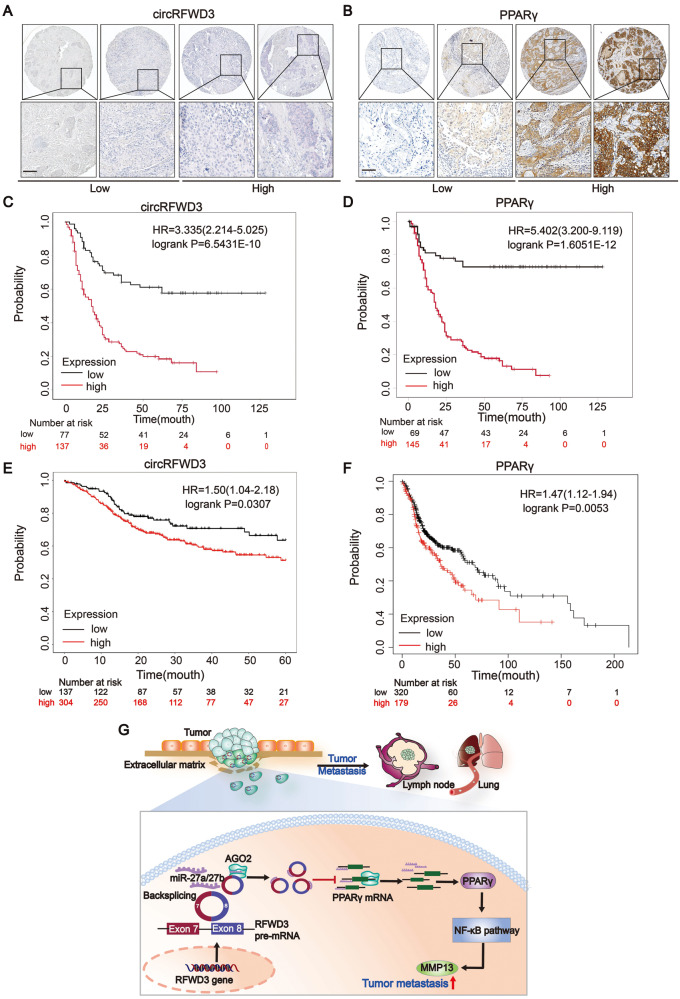
The expression of circRFWD3 and PPARγ were negatively associated with prognosis in patients with HNSCC. **A**, **B** ISH staining of circRFWD3(pink stain in the cytoplasm) and IHC staining of PPARγ in an HNSCC clinical cohort, scale bar, 10 μm. **C**, **D** Kaplan–Meier analysis showed that circRFWD3 and PPARγ were negatively correlated with the overall survival rate of HNSCC patients according to our clinical cohort. **E**, **F** Kaplan–Meier analysis showed that circRFWD3 and the mRNA expression of PPARγ were negatively correlated with the overall survival rate of HNSCC patients in the TCGA database. **G** Molecular mechanism of circRFWD3 involved in HNSCC metastasis. CircRFWD3, resulting from back-splicing of exons 7 and 8, could sponge-like bind to miR-27a/b to relieve the inhibitory effect of miR-27a/b on PPARγ, promoting the transcription and translation of PPARγ. Then, upregulated PPARγ could activate the NF-κB signaling pathway to promote the expression of MMP13 and accelerate tumor metastasis.

Using an HNSCC tissue microarray (TMA) consisting of 214 HNSCC tissues, we assessed the correlation of circRFWD3 or PPARγ expression with clinicopathological characteristics by ISH or IHC (Fig. 7A, B). Further analysis showed that the expression levels of both circRFWD3 and PPARγ were significantly correlated with lymph node metastasis (*P* = 0.013 and *P* = 0.004, respectively, Tables 1, 2) of HNSCC. PPARγ expression was also positively correlated with the age (*P* = 0.038) and clinical TNM stage (*P* = 0.000) of HNSCC (Table 2). Furthermore, Kaplan–Meier analysis demonstrated that HNSCC patients with low expression levels of circRFWD3 or protein expression levels of PPARγ had a longer OS than those with high expression (*P* < 0.0001, Fig. 7C, D). Consistently, both the expression of circRFWD3 and the mRNA expression of PPARγ were negatively correlated with the overall survival of HNSCC patients from the TCGA database (*P* = 0.0307 and *P* = 0.0053, respectively, Fig. 7E, F). Therefore, circRFWD3 and PPARγ could serve as tumor promoters, and their high expression might predict a poor prognosis for HNSCC patients. These findings suggested that circRFWD3 and PPARγ could be potential targets for predicting the prognosis of patients with HNSCC.

**Table 1:** Correlation of circRFWD3 in HNSCC with clinicopathological factors including patients and tumor characteristics.

Characteristics	Number of Cases	CircRFWD3 (Low)	CircRFWD3 (High)	*p* value^a^
Age at surgery				
≤60 years	94	33	61	
60 years	120	44	76	0.932
Gender				
Male	154	46	108	
Female	60	31	29	0.819
Smoking				
No	102	43	59	
Yes	112	34	78	0.292
Alcohol consumption				
No	105	44	61	
Yes	109	33	76	0.340
Differentiation				
High	136	56	80	
Moderate	56	17	39	
Poor	22	4	18	
High vs. moderate + poor				0.085
Tumor stage				
T1	48	14	34	
T2	103	42	61	
T3	20	8	12	
T4	43	13	30	
T1-2 vs. T3-4				0.105
Clinical TNM stage				
I	32	10	22	
II	70	31	39	
III	67	21	46	
IV	45	15	30	
I–III vs IV				0.333
Lymph node metastasis				
No	137	56	81	
Unilateral	70	20	50	
Bilateral	7	1	6	
No vs. Unilateral + Bilateral				0.013

^a^*P values* were derived using the Spearman rank correlation coefficient test; all statistical tests are two-sided.

The expression levels of circRFWD3 were significantly correlated with lymph node metastasis (*P* = 0.013) of HNSCC according to our clinical cohort (214 cases of HNSCC).

**Table 2:** Correlation of PPARγ in HNSCC with clinicopathological factors including patients and tumor characteristics.

Characteristics	Number of Cases	PPARγ (Low)	PPARγ(High)	*p* value^a^
Age at surgery				
≤60 years	94	29	65	
60 years	120	40	80	0.038
Gender				
Male	154	51	103	
Female	60	18	42	0.900
Smoking				
No	102	30	72	
Yes	112	39	73	0.866
Alcohol consumption				
No	105	34	71	
Yes	109	35	74	0.585
Differentiation				
High	136	46	90	
Moderate	56	20	36	
Poor	22	3	19	
High vs. moderate + poor				0.304
Tumor stage				
T1	48	14	34	
T2	103	36	67	
T3	20	4	16	
T4	43	15	28	
T1-2 vs. T3-4				0.000
Clinical TNM stage				
I	32	8	24	
II	70	25	45	
III	67	23	44	
IV	45	13	32	
I–III vs IV				0.955
Lymph node metastasis				
No	137	47	90	
Unilateral	70	21	49	
Bilateral	7	1	6	
No vs. unilateral + bilateral				0.004

^a^*P values* were derived using the Spearman rank correlation coefficient test; all statistical tests are two-sided.

The expression levels of PPARγ were significantly correlated with lymph node metastasis (*P* = 0.004), the age (*P* = 0.038), and clinical TNM stage (*P* = 0.000) of HNSCC according to our clinical cohort (214 cases of HNSCC).

## Discussion

HNSCC is the sixth most common malignant tumor in the world. The incidence is increasing year by year, and the prognosis is poor. Metastasis is one of the main reasons for the poor prognosis of HNSCC. However, due to the lack of effective therapeutic targets, HNSCC metastasis-targeted therapy has not yet made a major breakthrough. Therefore, it is urgent to elucidate the regulatory mechanism of HNSCC metastasis and discover valuable therapeutic targets. Here, we discovered a novel mechanism in HNSCC metastasis: circRFWD3 could sponge miR-27a/b to promote metastasis by augmenting PPARγ/NF-κB/MMP13 signaling (Fig. 7G).

CircRNAs, as a new type of noncoding RNAs, mainly have several characteristics: species conservation, unique circular and stable structures, cell and tissue specificity, and stable expression in saliva, blood, and extracellular vesicles. Increasing amounts of circRNAs have been detected and their biogenesis, characteristics, and functions have been explored in a variety of cancers [23, 24]. To date, despite rapid advances in studies based on circRNAs, little is known about the explicit roles of circRNAs in HNSCC, especially in the development and metastasis of HNSCC [25, 26]. In this study, we reported comprehensive circRNA expression profiles of HNSCC. According to our analysis and summary, a large number of circRNAs are abnormally expressed, among them, circRFWD3 was selected and characterized and exhibited an important function in regulating HNSCC metastasis.

CircRFWD3 is derived from the RFWD3 gene, which has a susceptible site for malignant neoplasms [27, 28]. In our study, we demonstrated that circRFWD3 originating from RFWD3 was abnormally overexpressed in HNSCC, and weakening circRFWD3 could inhibit the athletic ability of HNSCC cells. The most well-known function of circRNA is miRNA sponge in cancers [29]. Here, we identified that circRFWD3 contained conserved sites for two members of the miR27 family, miR-27a and miR-27b. Previous studies have reported the dual effect of cancer-promoting and cancer-inhibiting roles played by miR-27a and miR-27b in different cancers [30, 31]. Compared with previous findings, we reported for the first time that circRFWD3 exerts its functions in promoting tumor metastasis by harboring miR-27a/b to enhance the expression of PPARγ, which is the downstream target of miR-27a/b.

PPARγ is a member of the peroxisome proliferator-activated receptors, and its famous function is to regulate fat cell differentiation, body immunity, and insulin resistance [32]. The role of PPARγ in tumor development is controversial. On the one hand, on account of its proapoptotic and antiproliferative functions, PPARγ has been extensively considered a cancer suppressor, such as in colorectal cancer and breast cancer [33]. On the other hand, increasing evidence indicates that PPARγ acts as a cancer promoter. For example, PPARγ was reported to induce the production of reactive oxygen species to promote tumor growth in glioblastoma [34]. Different roles of PPARγ in different tumors, which may be due to organ-specific, considering that HNSCC also have organ specificity, we wanted to clarify the role of this molecule in HNSCC. In this study, we proved that PPARγ is negatively correlated with HNSCC prognosis and could promote metastasis of HNSCC, and the inhibition of endogenous PPARγ by reducing circRFWD3 or increasing miR-27a/b expression could restrain the migration and invasion ability of tumors.

The NF-κB signaling pathway is activated in multiple types of cancers and could play a role as a tumorigenesis promotor [35]. Rel B and p65 are two core members of the NF-κB family. It was reported that NF-κB suppression is beneficial for inhibiting tumor growth and metastasis in HNSCC [36]. The protein family of matrix metalloproteinases can degrade extracellular matrix components, whose well-known role is modulating tumor metastases, such as MMP2, MMP9, MMP7, MMP13, and MMP14 [37]. Previous reports showed that MMP13 could regulate tumor aggressiveness in oral cancer via the HBP1-MMP13 axis and can act as a diagnostic and prognostic molecular marker in HNSCC [38]. Because NF-κB is one of the transcription factors of MMP13, it is supposed that PPARγ could regulate MMP13 through NF-κB. As hypothetically, we demonstrated that PPARγ could regulate the NF-κB/MMP13 pathway. Furthermore, suppression of circRFWD3 suppressed the NF-κB pathway and the expression of MMP13 in HNSCC cells, while miR-27a and miR-27b inhibitors reversed this suppression. The data suggested that circRFWD3 enhanced the migration and invasion ability of HNSCC by weakening the expression of miR-27a/b, upregulating PPARγ, and then activating the NF-κB/MMP13 signaling axis.

## Conclusion

In summary, our findings suggested the presence of the circRFWD3/miR-27a/b /PPARγ/NF-κB/MMP13 axis in regulating HNSCC metastasis, highlighting the potential of circRFWD3 as a novel therapeutic target in HNSCC metastasis. Furthermore, the clinical expression patterns of circRFWD3 and PPARγ suggested that they may be promising prognostic biomarkers and therapeutic targets for HNSCC.

## Methods

### Patients and follow-up

A total of 9 pairs of tumors and adjacent nontumor tissues and 30 cases of metastatic and nonmetastatic samples were collected from pathologically diagnosed patients with HNSCC (Appendix Table S1). Noteworthily, a cohort of 214 HNSCC pathological samples and related clinical information was obtained (Tables 1, 2). Data on the other cohort comprising 499 patients with HNSCC, were obtained from The Cancer Genome Atlas (TCGA), EGA, and GEO database. The study was approved by the ethics committees of the West China Hospital of Stomatology, Sichuan University, and was conducted in agreement with the Helsinki Declaration. Written informed consent was provided by all participants at baseline and during follow-up.

### CircRNA microarray analysis

Nine tumor tissues were divided into groups of three and paired adjacent nontumor tissues were also divided into corresponding groups of three. Then, a total of six groups of tissues were deployed for circRNA microarray (Arraystar Inc.) and analyzed using Arraystar Human circRNA Array V2. Raw sequencing data of circRNAs have been uploaded to GEO(GSE200946). Overall, the assay indicated a total of 3157 circRNAs, Among them, 59 circRNAs were stably upregulated and 76 circRNAs were stably downregulated in the three groups in comparison with nontumor tissues. Then, we screened the candidates according to the following strategies: 200 bp ≤ the length of the base ≤ 1500; fold change ≥2 and *P* < 0.05. After validation in circBase, 32 circRNAs were obtained (Appendix Table S2).

### Cell cultures

Detailed information on HOK, UM1, UM2, HSC-3, CAL27, HN12, HN31, HN30, H413, and HEK293T cells is shown in Appendix Table S3. HOK cells were cultured in a defined keratinocyte SFM medium (10744019, Thermo Fisher Scientific). UM1, UM2, HSC-3, CAL27, HN12, HN31, H413, and HEK293T cells were appropriately cultured in DMEM (SH30243.01, HyClone) supplemented with 10% FBS and 1% penicillin-streptavidin solution. All cells were regularly tested for mycoplasma with a MycAwayTM Plus-Color One-Step Mycoplasma Detection Kit (40612ES25, Yeasen). Cells were kept in a humidified incubator at 37 °C with 5% CO_2_.

### Oligonucleotide transfection

Six-well plates were used to culture UM1 and HN31 cells, and transduction was carried out when cells were 70–80% confluent. Serum-free medium was used before transduction reagents were added to the corresponding plates. Cells were transfected using Lipofectamine 2000 (11668019, Invitrogen Lipofectamine 2000) following the product’s manual, and cells were seeded after being cultured for 48–72 h. All siRNAs targeting circRFWD3 and miRNA mimics or inhibitors were synthesized by RiboBio (Guangzhou, China).

### Plasmids

An expression construct encoding shRNA of circRFWD3 was cloned into GV341plasmad to produce GV341-sh-circRFWD3 recombinant lentivirus (sh-circRFWD3) constructs, and empty GV341 lentivirus (sh-NC) was used as a control. The pmiR-27a/b-Luc plasmid was constructed by amplification of the upstream 2000 bp to downstream 100 bp sequence of miR-27a/b and subcloning into the pGL3-Basic plasmid. The wild-type or point-mutated 3′UTR of PPARγ reporter constructs (WT UTR or Mut UTR) were designed by adding a sequence of wild-type or point-mutated 3′UTR of PPARγ to the pGL3-Basic plasmid.

### Stable cell line generation

GV341-sh-circRFWD3 recombinant lentiviruses (sh-circRFWD3 and sh-NC) were purchased from NeuronBiotech (Shanghai Genechem Co., Ltd.). UM1 and HN31 cells were infected with sh-circRFWD3 and sh-NC. Selective culture medium containing 1 µg/ml puromycin was used to select the cells with stable expression of low circRFWD3 or vector controls. The expression of circRFWD3 was detected by RT-qPCR.

### RNA isolation and qRT-PCR

Total RNA was isolated using RNA Pure kits (TR-205-50, ZYMO RESEARCH) according to the manufacturer’s guidelines from either cultured cells or liquid nitrogen–frozen tissues. The concentration and purity of each RNA extract were checked at a 260/280 ratio on a NanoDrop Microvolume UV-Vis Spectrophotometer instrument (Thermo Fisher Scientific, One^c^). Complementary DNA (cDNA) synthesis was achieved by using the PrimeScript^TM^ RT Reagent Kit (RR037A, TaKaRa) according to the manufacturer’s guidelines. Quantitative PCR was performed with SYBR™ Select Master Mix (4472908, Applied Biosystems) following the product’s instructions. Primer sequences were listed in Appendix Table S4. Three technical replicates were set for every single reaction to ensure reliability and validity. The ΔΔCT value of the target gene expression was measured and assessed against the value of the reference genes GAPDH and U6.

### RNase R digestion treatment and Sanger sequencing

RNase R digestion treatment was performed by incubating 4 μg of total RNA mixture at 37 °C for 10 min with or without 3 U/μg of RNase R, as suggested by the product’s manual (RNR 07250, Epicenter). Following the removal of linear forms, qRT-PCR qualification was performed to confirm the circular structure of circRFWD3. Sanger sequencing covering the back-splicing junction sequence was performed using the qRT-PCR product of circRFWD3 by TSINGKE company. A total of 2% agarose gel was used for DNA electrophoresis. The sequences of divergent primers and convergent primers are shown in Appendix Table S4.

### Actinomycin D assay

UM1 and HN31 cells were exposed to a complete medium (DMEM with 1 μg/mL actinomycin D (A1410, Sigma-Aldrich) to block transcription for 0, 2, 4, 6, 8, 10, and 12 h. The cells were harvested, and the expression of circRFWD3 and RFWD3 mRNA was analyzed using qRT-PCR.

### Cell invasion assay

UM1 and HN31 cells at a concentration of 1 × 10^5^ cells in 200 μl of serum-free medium were inoculated in the upper chamber and coated with growth factor-reduced Matrigel^®^ (356234, Corning), and a medium containing 15% FBS was added to the lower chamber as a chemoattractant. After incubation for 24 h (UM1)/30 h (HN31), cells on the upper surface of the membrane and cells were removed by wiping with a Q-tip, and the invaded or migrated cells were fixed with formaldehyde and stained using 0.5% crystal violet. The numbers of migrated and invaded cells were counted in five randomly selected fields under a microscope. The experiment was replicated three times.

### Cell migration assay

The assay was similar to the in vitro invasion assay, except that no growth factor-reduced Matrigel^®^ (356,234, Corning) was added to the chamber. After incubation for 18 h (UM1) or 24 h (HN31), cells on the upper surface of cells were removed by wiping with a Q-tip, and the invaded or migrated cells were fixed with formaldehyde and stained using 0.5% crystal violet. Cells in five randomized fields of view at 400× were counted and represented as the average number of cells per field of view. The experiment was also replicated three times.

### RNA FISH

RNA fluorescence in situ hybridization (FISH) was performed using the Fluorescent in situ Hybridization Kit (lnc1CM001, RiboBio) according to the manufacturer’s instructions. Cy3-labeled probes targeting circRFWD3, 5FAM-labeled miR-27a/27b, and Cy3-labeled probes targeting PPARγ were synthesized by RiboBio (Guangzhou, China). Fluorescence was recorded with a confocal laser scanning microscope (FV3000, OLYMPUS).

### RNA pull-down assay

RNA pull-down assays were performed according to the Pierce™ Magnetic RNA-Protein Pull-Down Kit (20164, Thermo Scientific). Biotinylated circRFWD3, miR-27a, and miR-27b probes were synthesized by RiboBio (Guangzhou, China). Pull-down RNA released from the beads after cleansing was evaluated by qRT-PCR.

### RNA immunoprecipitation

RNA immunoprecipitation (RIP) experiments were performed with a RIP-Assay Kit (RN1001, BML) according to the manufacturer’s guidelines. Briefly, UM1 cells were collected and resuspended in 200 μL RIP lysis buffer. A total of 100 μL of each cell lysate was incubated with Protein G Agarose Beads (37478 S, Cell Signaling Technology) conjugated with 15 µl argonaute 2 (Anti-EIF2C2 (AGO2) (Human) mAb, RN003 M, BML) or control rabbit IgG protein with gentle agitation at 4 °C overnight. Immunoprecipitated RNA was then purified and measured by qRT-PCR for enrichment.

### Western blot analysis

For Western blot analysis, cells were washed three times with 1x PBS and then used for extraction of total proteins. Protein extracts were prepared by mammalian lysis buffer. Protein concentrations were measured by the Pierce™ BCA Protein Assay Kit (23250, Thermo Scientific). Protein extracts were separated by 10% SDS PAGE and transferred onto polyvinylidene fluoride (PVDF) membranes (Millipore, IPVH00010). Then, the PVDF membranes were blotted individually with appropriate primary antibodies against GAPDH (2148 S, Cell Signaling Technology, dilution rate 1:3000), PPARγ (2435 S, Cell Signaling Technology, dilution rate 1:1000), p65 (8242 S, Cell Signaling Technology, dilution rate 1:1000), p-p65 (3033 S, Cell Signaling Technology, dilution rate 1:1000), Rel B (ab33917, Abcam, dilution rate 1:1000), MMP13 (ab51072, Abcam, dilution rate 1:1000), MMP2 (40994 S, Cell Signaling Technology, dilution rate 1:1000), MMP7 (3801 S, Cell Signaling Technology, dilution rate 1:1000), and appropriate secondary antibodies (mouse, ZB-2305; rabbit, ZB2301, ZSGB-BIO, dilution rate 1:3000). Protein bands were visualized using a chemiluminescence system (Amersham Imager 600).

### Luciferase reporter assay

Wild-type and predicted binding site-specific mutated miR-27a, miR-27b, and PPARγ 3′ UTR sequences were synthesized and integrated into psiCHECK 2.0 vectors and cotransfected into HEK293T cells with miR-27a or miR-27b mimics using Lipofectamine 2000 (11668019, Invitrogen Lipofectamine 2000). HEK293T cells were lysed, and luciferase intensity was measured 24 h after transfection. The ratio of Renilla luciferase to firefly luciferase absorbance was calculated to quantitatively analyze the interaction between miR-27a and miR-27b and PPARγ using the luciferase reporter assay protocol recommended by Promega (E1910, Promega, USA).

### In situ hybridization (ISH)

ISH experiments were performed with an ISH-Assay Kit (323900, BaseScope Reagent Kit v2-RED) according to the manufacturer’s guidelines. The circRFWD3 detection probe sequence (BaseScope Probe-BA-Hs-RFWD3-E8E7-circRNA-Junc) was 5′-TTCCCTACTGAAGGAACAGATGCTAAGGAAACAGGCCGAGTTAGAATCAGCACAGTGCCGACTCCAACTGCAGGTCCTCACTGATAAGTGCACTAGGCTTCAAAGGCGTGTTCAGGACTTGCAAAAACTTACGTCACATCAAAGTCAGAATTTACAGCAACCCAGGGGCTCCCAAGCATGGGTCCTGAGCTGCTCACCCTCCAGCCAGGGCCAGCACAAGCACAAGTACCACTTCCAAAAGACCTTCACAGTATCTCAGGCAGGAAACTGCCGGATCATGGCATACTGTGATGCTCTGAGCTGCCTGGTGATATCACAGCCTTCTCCTCAGGCCTCTTTTCTTCCAG-3′. The circRFWD3 probe was diluted with diluent to 1%.

### Immunohistochemistry (IHC) analysis

Immunohistochemistry (IHC) for PPARγ (#2435 S, Cell Signaling Technology, dilution rate 1:100) was performed in FFPE specimens after antigen retrieval with citrate buffer (0.01 M, pH 6.0), and then it was visualized by diaminobenzidine (GK600510, DAKO 1:50 dilution). For PPARγ, the intensity of staining (0, no staining; 1, weakly stained; 2, moderately stained; 3, strongly stained) was assessed by two experienced pathologists without any knowledge of the clinical and pathological data, and the percentage of positive cells was noted. For the statistics of prognostic value in the HNSCC cohort, the total staining scale was divided into two categories: low expression (the staining intensity was 0 and 1) and high expression (the staining intensity was 2 and 3).

### mRNA expression analysis

Global human mRNA expression was analyzed by RNA sequencing (Novogene) following the manufacturer’s standard protocol. Briefly, after treatment with 20 μM GW9662, UM1 and HN31 cells were lysed with TRIzol reagent (15596026, Thermo Fisher), and total RNA was extracted according to the standard method. Genes with |fold change | ≥2 and *P* < 0.05) were considered differentially expressed. Next, RNA sequencing was performed, and target genes were analyzed.

### Animal experiments

Forty-eight female BALB/c nude mice (SPF level) aged 5–6 weeks were purchased from Charles River Company (Beijing, China), which were raised and manipulated in the Animal Central Laboratory of West China Second Hospital in compliance with the institutional guidelines for animal use and care. Mice were randomly divided into four groups: UM1 sh-circRFWD3, UM1 sh-NC, HN31 sh-circRFWD3, and HN31 sh-NC. Each group contains 12 mice (*n* = 12). Specifically, sh-circRFWD or sh-NC-infected UM1 and HN31 cells (Appendix Fig. 1C–F) were intravenously injected into the tail vein of mice to construct the lung metastasis mouse model. Caudal vein injection was performed with 100 μL stroke-physiological saline solution suspension of 10^6^ cells using a 1 mL insulin syringe. After injection, two mice died in 24 h because of cardiac failure and were excluded. Animal weight was monitored weekly following injection. Euthanasia was initiated by CO_2_ suffocation and finished by cervical dislocation. The time point for sacrifice was based on a combination of two criteria: loss of >15% of pre-injection body weight and/or when the mice showed signs of pain and distress. Following sacrifice, the lung tumor nodules were counted. The final sample numbers collected were UM1 sh-NC group (*n* = 11), UM1 sh-cirCRFWD3 group (*n* = 11), HN31 sh-NC group (*n* = 12), and HN31 sh-circRFWD3 group (*n* = 12). Then, hematoxylin-eosin (H&E) and IHC with anti-mitochondrial antibodies (ab92824, Abcam) were used to determine the pulmonary metastasis of nude mice in each group.

### H&E staining

Tissues were immersed in 4% paraformaldehyde for 24 h and washed overnight with running water. The tissue was then placed in an automatic dehydrator. After dehydration, the tissues were embedded in paraffin and sectioned at a thickness of 5 μm. Dewaxing and gradient alcohol hydration were conducted before the sections were counterstained with hematoxylin and eosin. After dehydration with an alcohol gradient, the sections were sealed with neutral gum.

### Statistical analysis

All statistical analyses were carried out using GraphPad Prism version 8.0. Means ± SD were presented in quantification bar graphs. The χ^2^ test was used to assess the statistical significance of the association of the expression of circRFWD3 and PPARγ with the patient’s clinicopathologic parameters. Comparisons between groups for statistical significance were performed with the independent-sample *t*-test. Correlations were analyzed by the Pearson correlation test. Survival analysis was performed by Kaplan–Meier curves and log-rank test for significance. *P* values of <0.05 were considered statistically significant. The data were the mean ± SD of three experiments. (*P < 0.05, **P < 0.01, ***P < 0.001).

## Supplementary information

Appendix file of circRFWD3 with original western blots

## Data Availability

All data generated or analysed during this study are included in this published article and its supplementary information files.
