# Correction: CircRFWD3 promotes HNSCC metastasis by modulating miR-27a/b/PPARγ signaling

**DOI:** 10.1038/s41420-025-02547-0

**Published:** 2025-07-30

**Authors:** Zihao Wei, Ying Wang, Jiakuan Peng, Honglin Li, Junjie Gu, Ning Ji, Taiwei Li, Xikun Zhou, Xin Zeng, Jing Li, Qianming Chen

**Affiliations:** 1 State Key Laboratory of Oral Diseases, National Clinical Research Center for Oral Diseases, Chinese Academy of Medical Sciences Research Unit of Oral Carcinogenesis and Management, West China Hospital of Stomatology, Sichuan University, Chengdu, Sichuan 610041 P.R. China; 2 State Key Laboratory of Biotherapy and Cancer Center, West China Hospital, Sichuan University and Collaborative Innovation Center for Biotherapy, Chengdu, 610041 China

Correction to: *Cell Death Discovery* 10.1038/s41420-022-01066-6, published online 11 June 2022

Two pictures have been found to be misplaced during the assembling. The first place is the Western blot band of Rel-B in UM1 cells and the Western blot band of MMP13 in both UM1 and HN31 cells of Fig. 6F. And another one is the Western blot band of MMP2 in UM1 and HN31 cells of Appendix Fig. 3E. Besides, we also found the molecular weights of Rel-B in Fig. 6E, Fig. 6F and Appendix Fig. 3F were mislabeled. In addition, Regarding editorial policies comment that “cropped gels in the paper must retain important bands,” we have replaced the gel images in Figure 6A (PPARγ), Figure 6E (UM1 PPARγ), Figure 6F (UM1 PPARγ), and Figure 6F (HN31 p65). The revised Fig.6 and Appendix Fig.3 are as follows.

**Amended Figure 6** 
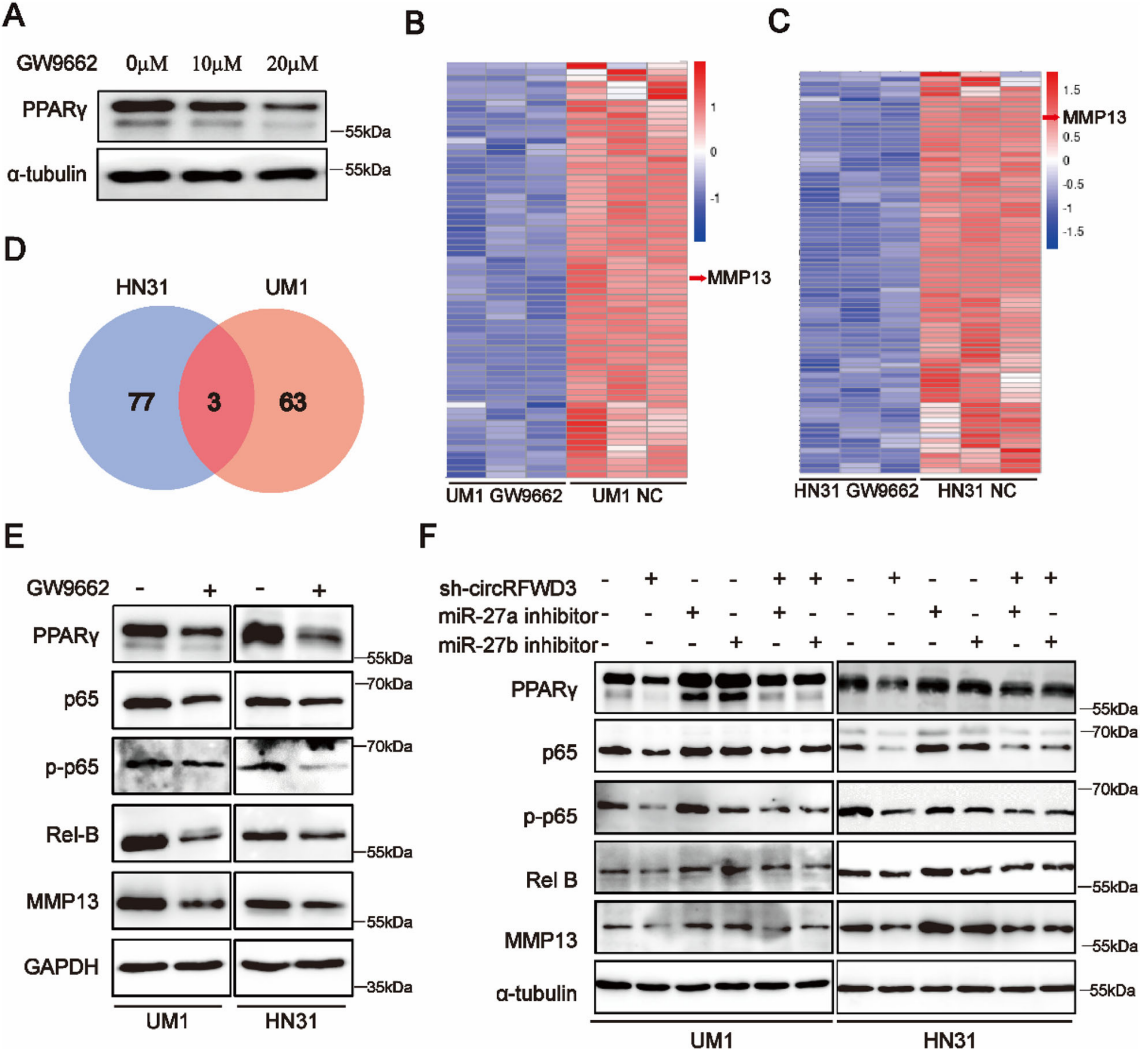


**Fig. 6** CircRFWD3 regulated PPARγ/NF-κB/MMP13 signal pathway via miR-27a/27b in HNSCC.

**Amended Supplementary Figure S3** 
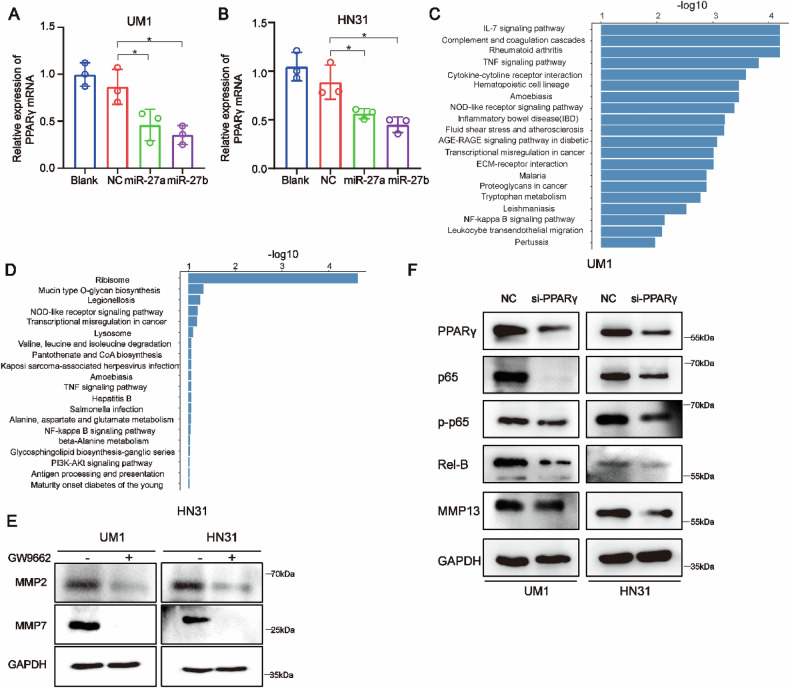


**Appendix Fig. 3** The downstream target gene of miR-27a/b and related signaling pathways in HNSCC.

## Appendix file of circRFWD3 with original western blots

Supplementary Information

