# Supplementary material for: CircRFWD3 promotes HNSCC metastasis by modulating miR-27a/b/PPARγ signaling
Source: Cell Death Discov. 2022 Jun 11;8:285. doi: 10.1038/s41420-022-01066-6 (PMC9188624; doi:10.1038/s41420-022-01066-6)

**CircRFWD3 promotes HNSCC metastasis by modulating miR-27a/b/PPARγ signaling**

Zihao Wei^1,#^, Ying Wang^1,#^, Jiakuan Peng^1^, Honglin Li^1^, Junjie Gu^1^, Ning Ji^1^, Taiwei Li^1^, Xikun Zhou^2^, Xin Zeng^1,^*, Jing Li^1,^*, Qianming Chen^1^


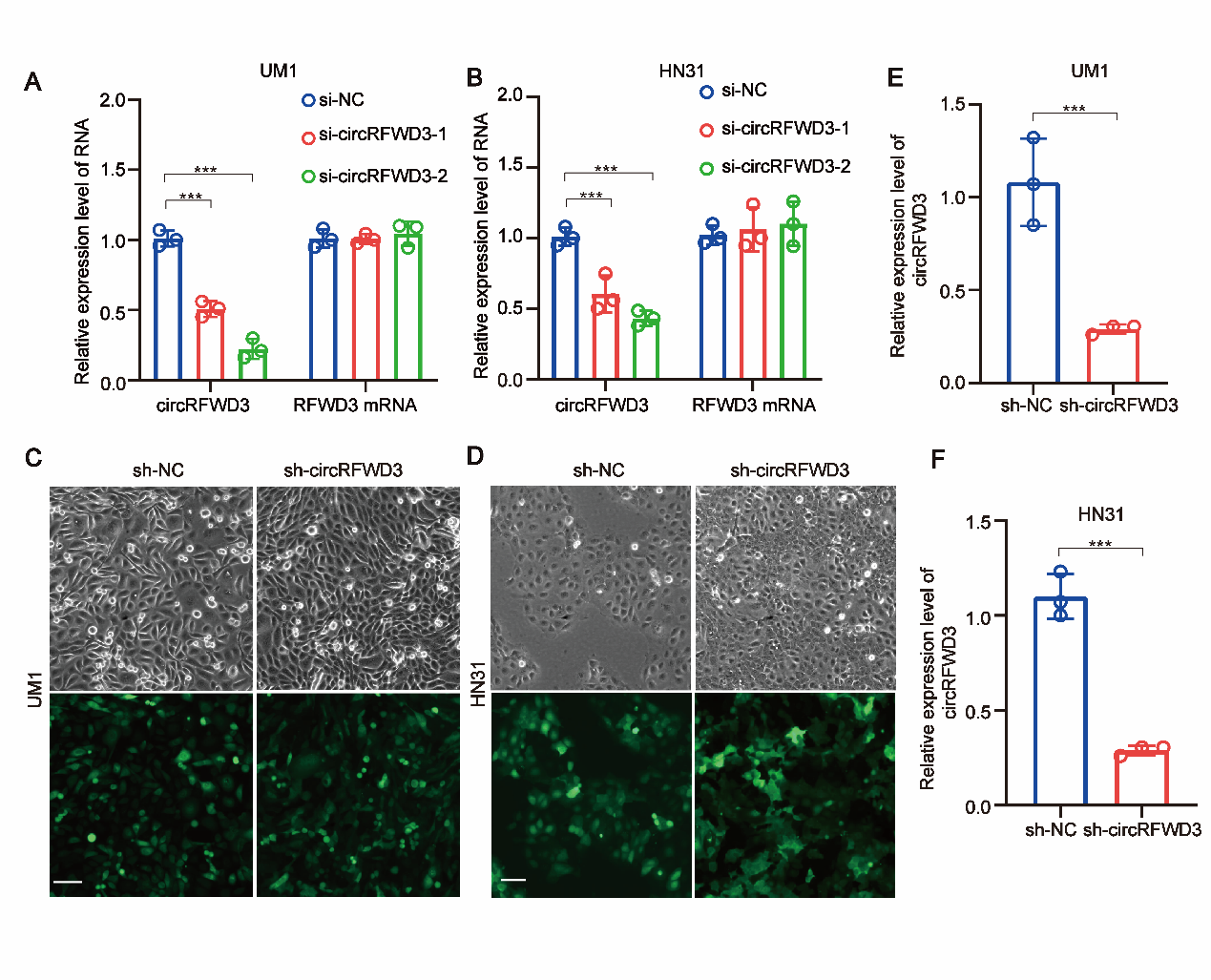


**Appendix Fig. 1: Construction of HNSCC cell lines with stable circRFWD3 knockdown.**

**(A, B)** Inhibitory efficiency of si-circRFWD3 in UM1 and HN31 cell lines evaluated by qRT-PCR. **(C, D)** Fluorescence microscopy showed that the fluorescent plasmid was successfully integrated into the UM1 and HN31 cell genomes. **(E, F)** qRT-PCR results showed that the expression of circRFWD3 was downregulated in sh-circRFWD3-transfected UM1 and HN31 cell lines. Scale bar, 100μm. Data are shown as the mean value ± SD of three independent experiments. The asterisks indicate significant differences (Student’s t-tests, ***P*＜0.01, ****P*＜0.001).

**
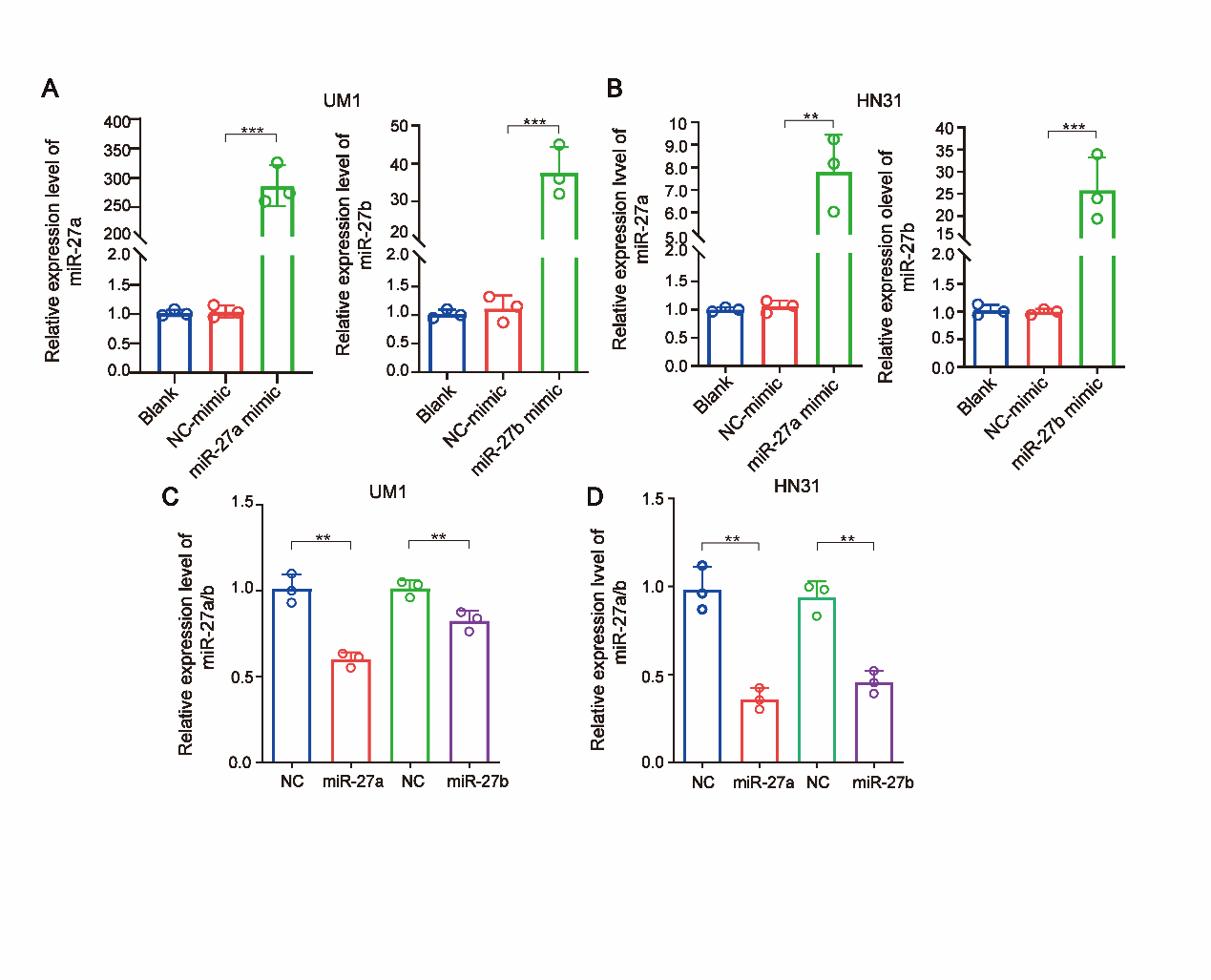
**

**Appendix Fig. 2**: **Enhancement and inhibitory efficiency of miR-27a/27b mimic and inhibitors.**

**(A, B)** Enhancement efficiency of miR-27a and miR-27b mimic in UM1 and HN31 cell lines evaluated by qRT-PCR. **(C, D)** Inhibitory efficiency of miR-27a and miR-27b inhibitors in UM1 and HN31 cell lines evaluated by qRT-PCR. Data are shown as the mean value ± SD of three independent experiments. The asterisks indicate significant differences (Student’s t-tests, **P*＜0.5, ***P*＜0.01, ****P*＜0.001).


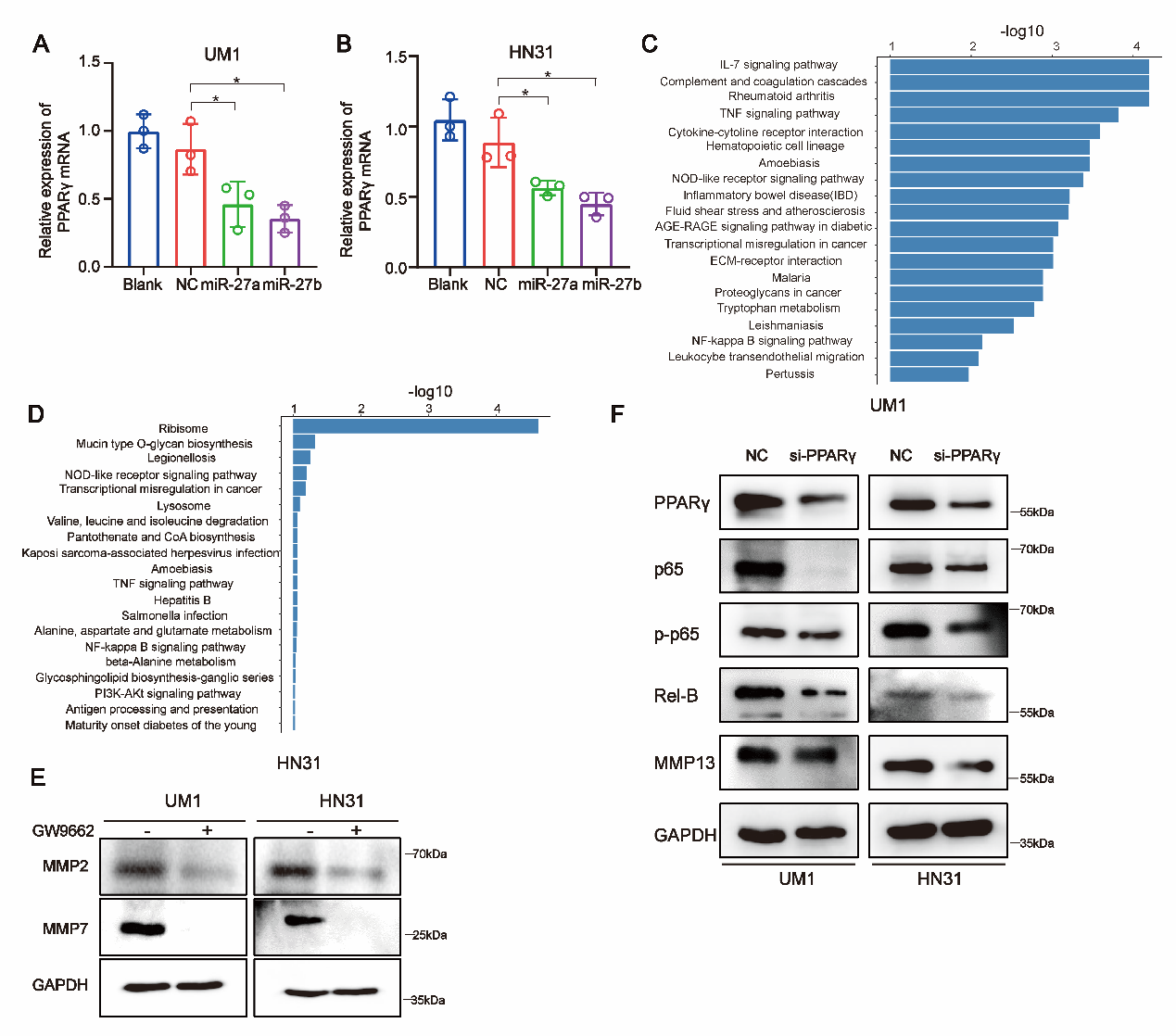


**Appendix Fig. 3: The downstream target gene of miR-27a/b and related signaling pathways in HNSCC**

**(A, B,)** qPCR assays showed that miR-27a and miR-27b mimic could significantly reduce the expression of PPARγ in UM1 and HN31 cells at the mRNA level. **(C, D)** Pathway enrichment analysis in GW9662-treated HNSCC lines. **(E)** Western blot analysis showed that inhibition of PPARγ decreased the expression levels of MMP2 and MMP7. **(F)** Western blot analysis showed that inhibition of PPARγ by siRNA decreased the expression levels of PPARγ, p65, p-P65, Rel B and MMP13. Data are shown as the mean value ± SD of three independent experiments. The asterisks indicate significant differences (Student’s t-tests, *P＜0.05, **P＜0.01, ***P＜0.001).

**Table S1. Characteristic of patients in the HNSCC clinical cohort**

| **characteristic** | |  | **N=30，Case（%）** | ***P Value*** |
| --- | --- | --- | --- | --- |
| **Gender** |  | |  |  |
|  | Male | | 20（66.7%） | 0.201 |
|  | Female | | 10（33.3%） |  |
| **Age** |  | |  |  |
|  | ＜60 years old | | 13（43.3%） | 0.584 |
|  | ≥60 years old | | 17（56.7%） |  |
| **Smoke** |  | |  |  |
|  | No | | 12（40%） | 1.000 |
|  | Yes | | 18（60%） |  |
| **Alcohol** |  | |  |  |
|  | No | | 14（46.7%） | 0.584 |
|  | Yes | | 16（53.3%） |  |
| **Clinical stages** |  | |  |  |
|  | I-II | | 8（26.7%） | 0.018 |
|  | III-IV | | 22（73.3%） |  |
| **Lymphatic metastasis** |  | |  |  |
|  | N0 | | 15（50%） | 0.855 |
|  | Nx | | 15（50%） |  |

**Table S2. Normalized intensity of 32 candidate circRNAs**

|  |  |  | **Normalized Intensity** | |  |  |
| --- | --- | --- | --- | --- | --- | --- |
| **circRNA** | **TA** | **CA** | **TB** | **CB** | **TC** | **CC** |
| **hsa_circRNA_400059** | **9.263587155** | **6.417325586** | **8.804030248** | **4.593081485** | **8.683469943** | **6.149112554** |
| **hsa_circRNA_400031** | **7.924627124** | **6.266201432** | **10.10182528** | **6.802651347** | **10.31057731** | **6.872033881** |
| **hsa_circRNA_104762** | **9.818798557** | **6.802651347** | **8.56085037** | **6.894439191** | **9.39689467** | **5.906139663** |
| **hsa_circRNA_104616** | **9.651294712** | **6.826151567** | **8.02865448** | **5.731602637** | **8.24500806** | **5.512082875** |
| **hsa_circRNA_102513** | **11.23788838** | **12.23788838** | **12.63044964** | **9.570112697** | **12.68496088** | **9.404010649** |
| **hsa_circRNA_101877** | **10.49831947** | **9.11972477** | **9.188923691** | **6.984537451** | **9.927361354** | **6.769975918** |
| **hsa_circRNA_001067** | **13.33375664** | **11.90098078** | **13.39327453** | **11.08998145** | **12.86656457** | **10.02958923** |
| **hsa_circRNA_400103** | **9.339223759** | **7.208336252** | **7.99181722** | **5.817250881** | **8.628635497** | **7.063957068** |
| **hsa_circRNA_104598** | **10.2636238** | **8.838842894** | **8.745289255** | **6.244166122** | **9.586781212** | **7.80285308** |
| **hsa_circRNA_104166** | **9.691252846** | **7.335855793** | **8.724691574** | **7.481547414** | **9.055649691** | **7.197728904** |
| **hsa_circRNA_101744** | **10.26577377** | **8.903787805** | **10.23018313** | **8.649143983** | **10.4566646** | **8.12223498** |
| **hsa_circRNA_100542** | **9.212217776** | **7.554349235** | **8.9952078** | **7.623043382** | **8.943400116** | **7.564546689** |
| **hsa_circRNA_103595** | **8.064125382** | **6.434107391** | **7.442684598** | **6.28328183** | **8.050585738** | **6.596966605** |
| **hsa_circRNA_100641** | **9.85974511** | **8.026811737** | **9.524807006** | **8.577940269** | **9.907344539** | **8.491059841** |
| **hsa_circRNA_104700** | **12.15760024** | **10.86738334** | **12.64643918** | **10.77900969** | **11.00653795** | **10.045262** |
| **hsa_circRNA_103401** | **9.560910009** | **7.798539483** | **8.983379699** | **8.0451341** | **9.028280287** | **7.71746242** |
| **hsa_circRNA_103563** | **13.16194049** | **11.97905671** | **13.56158256** | **12.69605515** | **13.88505782** | **12.24648393** |
| **hsa_circRNA_101711** | **10.84951101** | **9.788123589** | **10.85380501** | **9.637172072** | **10.65386207** | **9.365889584** |
| **hsa_circRNA_101379** | **9.727300106** | **11.08434476** | **7.490266149** | **9.086937942** | **8.139391612** | **9.383996785** |
| **hsa_circRNA_103915** | **6.076148121** | **7.898664531** | **6.076148121** | **7.898664531** | **5.784908163** | **6.68372608** |
| **hsa_circRNA_104067** | **6.195782842** | **7.348451783** | **6.240990565** | **7.341055702** | **5.961209143** | **7.10993974** |
| **hsa_circRNA_103704** | **6.857592631** | **7.946236336** | **5.767357588** | **7.264931495** | **5.413981389** | **6.18590838** |
| **hsa_circRNA_100783** | **7.876325256** | **9.378588447** | **7.858305012** | **8.649143983** | **7.520045897** | **8.306678951** |
| **hsa_circRNA_102285** | **8.138111918** | **9.470425918** | **6.802651347** | **7.570883689** | **7.34918931** | **7.995590669** |
| **hsa_circRNA_102347** | **6.631329058** | **7.688614774** | **6.631329058** | **7.688614774** | **6.763351076** | **7.338086621** |
| **hsa_circRNA_103917** | **6.40034627** | **7.490266149** | **6.894439191** | **7.51676723** | **6.429930205** | **7.191162328** |
| **hsa_circRNA_104236** | **8.628939214** | **9.759558857** | **7.30349563** | **8.146197542** | **7.631556226** | **8.113579542** |
| **hsa_circRNA_102634** | **7.132392906** | **7.667481386** | **5.999296012** | **6.961570597** | **6.287905238** | **7.393480079** |
| **hsa_circRNA_103188** | **14.03462436** | **12.77902891** | **14.24095046** | **13.30686421** | **14.53397667** | **13.17961919** |
| **hsa_circRNA_104503** | **12.0852961** | **10.76235611** | **11.77051336** | **10.35412312** | **11.50472074** | **9.617410119** |
| **hsa_circRNA_101740** | **8.345636375** | **6.651799561** | **8.20604786** | **7.320142657** | **8.265077924** | **6.240990565** |
| **hsa_circRNA_101852** | **11.14983312** | **8.990015379** | **9.423400521** | **8.479654084** | **10.84298049** | **9.232745753** |

**Table S3. The detailed information of cell cultures**

| **Cell Line** | **HOK** | **UM1** | **UM2** | **HSC-3** | **CAL27** | **HN12** | **HN30** | **HN31** | **H413** | **HEK293T** |
| --- | --- | --- | --- | --- | --- | --- | --- | --- | --- | --- |
| RRID | CVCL_B404 | CVCL_VH00 | CVCL_VH01 | CVCL_1288 | CVCL_1107 | CVCL_5518 | CVCL_5525 | CVCL_5526 | CVCL_2465 | CVCL_0063 |
| Species of origin | Homo sapiens  (Human)  (NCBI  Taxonomy:  9606) | Homo sapiens  (Human)  (NCBI  Taxonomy:  9606) | Homo sapiens  (Human)  (NCBI  Taxonomy:  9606) | Homo sapiens  (Human)  (NCBI  Taxonomy:  9606) | Homo sapiens (Human) (NCBI Taxonomy: 9606) | Homo sapiens (Human) (NCBI Taxonomy: 9606) | Homo sapiens (Human) (NCBI Taxonomy: 9606) | Homo sapiens (Human) (NCBI Taxonomy: 9606) | Homo sapiens (Human) (NCBI Taxonomy: 9606) | Homo sapiens (Human) (NCBI Taxonomy: 9606) |
| Sex of cell | Male | Male | Male | Male | Male | Female | Male | Male | Female | Female |
| Age at sampling | Age unspecified | 47Y | 47Y | 64Y | 56Y | Age unspecified | Age unspecified | Age unspecified | 53Y | Fetus |
| Disease | Human Oral Keratinocytes | Tongue squamous cell carcinoma (NCIt: C4648) | Tongue squamous cell carcinoma (NCIt: C4648) | Tongue squamous cell carcinoma (NCIt: C4648) | Tongue squamous cell carcinoma (NCIt: C4648) | Tongue squamous cell carcinoma (NCIt: C4648) | Pharyngeal squamous cell carcinoma (NCIt: C102872) | Pharyngeal squamous cell carcinoma (NCIt: C102872) | Buccal mucosa squamous cell carcinoma (NCIt: C4040) | NA |
| Derived from | Oral cavity; keratinocyte. | Primary lesion: Tongue | Primary lesion: Tongue | Metastatic site: Cervical lymph node | Primary lesion: Tongue | Derived from metastatic site: Lymph node. | Primary lesion: pharynx | Primary lesion: pharynx | Primary lesion: buccal mucosa. | Fetal kidney. |
| Website | https://web.expasy.org/cellosaurus/CVCL_B404 | https://web.expasy.org/cellosaurus/CVCL_VH00 | https://web.expasy.org/cellosaurus/CVCL_VH01 | https://web.expasy.org/cellosaurus/CVCL_1288 | https://web.expasy.org/cellosaurus/CVCL_1107 | https://web.expasy.org/cellosaurus/CVCL_5518 | https://web.expasy.org/cellosaurus/CVCL_5525 | https://web.expasy.org/cellosaurus/CVCL_5526 | https://web.expasy.org/cellosaurus/CVCL_2465 | https://web.expasy.org/cellosaurus/CVCL_0063 |

**Table S4. Primer information**

| **Gene** | **Forward primer (5’→3’)** | **Reverse primer (5’→3’)** |
| --- | --- | --- |
| GAPDH | ACATCGCTCAGACACCATG | TGTAGTTGAGGTCAATGAAGGG |
| U6 | CAGCACATATACTAAAATTGGAACG | ACGAATTTGCGTGTCATCC |
| RFWD3 | GCCTCTGCTACCTTCTGCTTC | AGCATTGGTCCACTGTTCCAG |
| circRFWD3 | TACTGTGATGCTCTGAGCTGCCTGGTG | CCTGAACACGCCTTTGAAGC |
| divergent primer | TGATGCTCTGAGCTGCCTGG | GAGCCCCTGGGTTGCTGTAA |
| convergent primer | TGCACTGGGGACATTTTCGT | AGTCTGAGCCTCTGCTACCT |

**Table S5. Upregulated and downregulated mRNAs in PPARγ-repressed UM1 cells**

| Gene_ID | UM1_  GW9662_1 | UM1_  GW9662_2 | UM1_  GW9662_3 | UM1_NC_1 | UM1_NC_2 | UM1_NC_3 |
| --- | --- | --- | --- | --- | --- | --- |
| ENSG00000169509 | 29.7885961 | 31.75619615 | 31.89967343 | 2.033316061 | 0 | 0.893275548 |
| ENSG00000110090 | 1856.467864 | 1869.766337 | 1836.224952 | 90.48256473 | 110.0611249 | 89.32755484 |
| ENSG00000167772 | 50.0022863 | 73.13548203 | 62.80248207 | 9.149922276 | 8.228869153 | 4.466377742 |
| ENSG00000162344 | 37.23574512 | 49.07775768 | 63.79934686 | 5.083290153 | 3.085825932 | 13.39913323 |
| ENSG00000164007 | 25.53308237 | 37.53004999 | 26.91534946 | 3.049974092 | 4.114434576 | 6.252928839 |
| ENSG00000171303 | 38.29962355 | 40.41697691 | 46.85264535 | 4.066632123 | 9.257477797 | 6.252928839 |
| ENSG00000176046 | 12.76654118 | 12.51001666 | 24.92161987 | 5.083290153 | 1.028608644 | 1.786551097 |
| ENSG00000108846 | 839.4000828 | 993.1028613 | 1085.585761 | 162.6652849 | 168.6918176 | 138.45771 |
| ENSG00000155918 | 24.46920394 | 15.39694359 | 25.91848466 | 4.066632123 | 2.057217288 | 4.466377742 |
| ENSG00000255150 | 22.34144707 | 14.43463461 | 10.96551274 | 3.049974092 | 4.114434576 | 0.893275548 |
| ENSG00000179148 | 43.61901571 | 42.34159486 | 38.87772699 | 8.133264246 | 5.14304322 | 9.826031033 |
| ENSG00000126878 | 13.83041962 | 18.28387051 | 15.94983671 | 2.033316061 | 3.085825932 | 4.466377742 |
| ENSG00000176153 | 140.431953 | 171.2909974 | 181.4293926 | 31.51639895 | 39.08712848 | 29.4780931 |
| ENSG00000069702 | 153.1984942 | 121.2509307 | 126.6018289 | 38.63300517 | 22.62939017 | 30.37136865 |
| ENSG00000099994 | 124.4737765 | 160.7055987 | 179.435663 | 45.74961138 | 28.80104203 | 32.15791974 |
| ENSG00000113739 | 1689.43895 | 2001.602666 | 2186.124495 | 458.5127718 | 426.8725873 | 465.3965607 |
| ENSG00000171658 | 621.3050043 | 524.4583909 | 653.9433053 | 137.2488341 | 130.6332978 | 146.4971899 |
| ENSG00000178821 | 36.17186669 | 41.37928589 | 43.86205097 | 7.116606215 | 14.40052102 | 7.146204387 |
| ENSG00000230397 | 29.7885961 | 16.35925256 | 15.94983671 | 7.116606215 | 6.171651865 | 1.786551097 |
| ENSG00000267480 | 42.55513728 | 44.26621281 | 42.86518617 | 7.116606215 | 10.28608644 | 14.29240877 |
| ENSG00000171401 | 22.34144707 | 22.1331064 | 23.92475507 | 2486.745543 | 2982.965068 | 3281.001089 |
| ENSG00000241494 | 1.063878432 | 1.924617948 | 2.990594384 | 5.083290153 | 14.40052102 | 210.8130294 |
| ENSG00000140379 | 1.063878432 | 0.962308974 | 0.996864795 | 23.38313471 | 17.48634695 | 16.07895987 |
| ENSG00000170477 | 1.063878432 | 0.962308974 | 0.996864795 | 8.133264246 | 28.80104203 | 16.07895987 |
| ENSG00000187054 | 1.063878432 | 4.811544871 | 0 | 28.46642486 | 22.62939017 | 50.91670626 |
| ENSG00000138131 | 18.08593334 | 9.623089741 | 9.968647947 | 187.0650776 | 208.8075548 | 198.3071717 |
| ENSG00000163209 | 2.127756864 | 1.924617948 | 0 | 8.133264246 | 25.7152161 | 18.75878652 |
| ENSG00000096996 | 2.127756864 | 0.962308974 | 1.993729589 | 33.54971501 | 16.45773831 | 8.932755484 |
| ENSG00000137868 | 13.83041962 | 9.623089741 | 16.94670151 | 169.7818911 | 162.5201658 | 129.5249545 |
| ENSG00000127954 | 31.91635296 | 25.02003333 | 26.91534946 | 347.6970465 | 204.6931202 | 338.5514328 |
| ENSG00000171346 | 5255.559454 | 6277.141438 | 7296.053432 | 53920.49197 | 59014.36374 | 55910.11658 |
| ENSG00000101144 | 0 | 5.773853845 | 2.990594384 | 28.46642486 | 25.7152161 | 25.01171536 |
| ENSG00000242611 | 3.191635296 | 0 | 6.978053563 | 33.54971501 | 26.74382475 | 23.22516426 |
| ENSG00000162692 | 5.31939216 | 0 | 2.990594384 | 19.31650258 | 23.65799881 | 24.11843981 |
| ENSG00000224689 | 4.255513728 | 4.811544871 | 1.993729589 | 16.26652849 | 31.88686797 | 41.98395078 |
| ENSG00000196611 | 36.17186669 | 52.92699358 | 27.91221425 | 274.4976683 | 312.6970278 | 355.5236683 |
| ENSG00000135960 | 5.31939216 | 6.736162819 | 3.987459179 | 47.78292744 | 41.14434576 | 40.19739968 |
| ENSG00000137673 | 4.255513728 | 9.623089741 | 2.990594384 | 35.58303107 | 37.02991119 | 63.42256394 |
| ENSG00000232480 | 4.255513728 | 10.58539872 | 3.987459179 | 56.93284972 | 26.74382475 | 66.10239058 |
| ENSG00000079337 | 55.32167846 | 75.06009998 | 74.7648596 | 531.7121501 | 496.8179751 | 495.7679294 |
| ENSG00000148344 | 70.21597651 | 119.3263128 | 99.68647947 | 682.1775386 | 759.1131793 | 594.0282397 |
| ENSG00000250328 | 3.191635296 | 4.811544871 | 0.996864795 | 15.24987046 | 14.40052102 | 33.05119529 |
| ENSG00000142619 | 2.127756864 | 8.660780767 | 4.984323973 | 43.71629532 | 37.02991119 | 30.37136865 |
| ENSG00000215874 | 1.063878432 | 2.886926922 | 3.987459179 | 12.19989637 | 19.54356424 | 23.22516426 |
| ENSG00000137745 | 1340.486824 | 1103.768393 | 826.4009148 | 6049.115283 | 6421.603765 | 9512.491315 |
| ENSG00000070808 | 109.5794785 | 85.64549869 | 88.72096673 | 720.8105438 | 523.5617998 | 659.2373547 |
| ENSG00000169248 | 4.255513728 | 5.773853845 | 5.981188768 | 40.66632123 | 32.91547661 | 33.94447084 |
| ENSG00000253522 | 3.191635296 | 4.811544871 | 4.984323973 | 22.36647668 | 23.65799881 | 40.19739968 |
| ENSG00000082074 | 78.72700397 | 58.70084742 | 73.76799481 | 433.0963211 | 393.9571107 | 562.7635955 |
| ENSG00000231483 | 2.127756864 | 4.811544871 | 3.987459179 | 22.36647668 | 15.42912966 | 32.15791974 |
| ENSG00000064300 | 185.1148472 | 276.1826756 | 336.9403006 | 1851.334274 | 1822.694517 | 1403.335887 |
| ENSG00000206073 | 28.72471766 | 39.45466794 | 40.87145658 | 266.364404 | 182.06373 | 235.8247448 |
| ENSG00000232325 | 19.14981178 | 17.32156153 | 16.94670151 | 132.165544 | 98.74642983 | 101.8334125 |
| ENSG00000162496 | 159.5817648 | 204.0095025 | 210.3384717 | 1265.739248 | 1248.730894 | 939.7258769 |
| ENSG00000168497 | 5.31939216 | 17.32156153 | 17.9435663 | 66.082772 | 83.31730017 | 83.96790155 |
| ENSG00000187689 | 5.31939216 | 3.849235896 | 2.990594384 | 22.36647668 | 20.57217288 | 25.01171536 |
| ENSG00000163435 | 185.1148472 | 232.8787717 | 262.175441 | 1206.773082 | 1424.622972 | 1155.89856 |
| ENSG00000162654 | 119.1543844 | 102.9670602 | 115.6363162 | 680.1442225 | 533.8478863 | 581.522382 |
| ENSG00000111181 | 2.127756864 | 1.924617948 | 4.984323973 | 21.34981864 | 16.45773831 | 9.826031033 |
| ENSG00000101846 | 11.70266275 | 2.886926922 | 1.993729589 | 32.53305698 | 26.74382475 | 24.11843981 |
| ENSG00000163220 | 1428.788734 | 2529.910293 | 1912.983541 | 8277.629686 | 9924.016198 | 10827.39292 |
| ENSG00000146666 | 7.447149024 | 3.849235896 | 4.984323973 | 22.36647668 | 20.57217288 | 36.62429748 |
| ENSG00000140465 | 40.42738042 | 51.00237563 | 34.89026781 | 226.7147408 | 180.0065127 | 212.5995805 |
| ENSG00000196104 | 4.255513728 | 8.660780767 | 2.990594384 | 26.4331088 | 23.65799881 | 27.691542 |
| ENSG00000115009 | 12.76654118 | 5.773853845 | 10.96551274 | 43.71629532 | 31.88686797 | 65.20911503 |
| ENSG00000167779 | 342.5688551 | 666.8801191 | 574.1941217 | 2509.11202 | 2648.667259 | 2456.507758 |
| ENSG00000250657 | 9.574905888 | 3.849235896 | 0 | 14.23321243 | 21.60078153 | 25.9049909 |
| ENSG00000167755 | 8.511027456 | 12.51001666 | 8.971783152 | 54.89953366 | 46.28738898 | 38.41084858 |
| ENSG00000124391 | 5.31939216 | 0.962308974 | 6.978053563 | 22.36647668 | 19.54356424 | 18.75878652 |
| ENSG00000134339 | 4.255513728 | 7.698471793 | 7.974918357 | 34.56637304 | 23.65799881 | 33.94447084 |
| ENSG00000185215 | 2238.400221 | 3107.295677 | 3710.330766 | 16349.89445 | 13461.40133 | 11184.70314 |
| ENSG00000104783 | 58.51331376 | 89.49473459 | 56.8212933 | 325.3305698 | 334.2978093 | 263.5162868 |
| ENSG00000178726 | 552.1529062 | 589.8954011 | 634.0060094 | 2919.841864 | 2744.327862 | 2201.030951 |
| ENSG00000205488 | 22.34144707 | 22.1331064 | 18.9404311 | 96.58251292 | 85.37451746 | 98.26031033 |
| ENSG00000205572 | 7.447149024 | 7.698471793 | 4.984323973 | 15.24987046 | 48.34460627 | 24.11843981 |
| ENSG00000183347 | 517.0449179 | 467.6821614 | 475.5045071 | 2079.065673 | 1889.554079 | 2339.488661 |
| ENSG00000140519 | 9.574905888 | 29.8315782 | 19.93729589 | 75.23269427 | 86.4031261 | 95.58048368 |
| ENSG00000183742 | 61.70494905 | 48.11544871 | 59.81188768 | 204.3482642 | 228.351119 | 285.8481755 |
| ENSG00000080031 | 9.574905888 | 10.58539872 | 14.95297192 | 47.78292744 | 58.63069271 | 42.87722632 |
| ENSG00000184254 | 108.5156001 | 161.6679076 | 158.5015024 | 620.1613987 | 599.6788395 | 576.1627287 |
| ENSG00000126233 | 4.255513728 | 3.849235896 | 5.981188768 | 25.41645077 | 14.40052102 | 18.75878652 |
| ENSG00000136689 | 35.10798826 | 61.58777434 | 58.81502289 | 219.5981346 | 210.864772 | 212.5995805 |
| ENSG00000057149 | 105.3239648 | 77.9470269 | 68.78367083 | 324.3139118 | 296.2392895 | 414.4798545 |
| ENSG00000116106 | 92.55742358 | 99.11782433 | 92.70842591 | 364.980233 | 363.0988514 | 420.7327833 |
| ENSG00000163739 | 204.2646589 | 218.4441371 | 221.3039844 | 919.0588598 | 805.4005683 | 877.1965885 |
| ENSG00000030419 | 89.36578829 | 67.36162819 | 62.80248207 | 258.2311398 | 271.552682 | 346.5909128 |

**Table S6. Upregulated and downregulated mRNAs in PPARγ-repressed HN31 cells**

| Gene_ID | HN31_GW9662_1 | HN31_GW9662_2 | HN31_GW9662_3 | HN31_NC_1 | HN31_NC_2 | HN31_NC_3 |
| --- | --- | --- | --- | --- | --- | --- |
| ENSG00000099994 | 468.9295913 | 503.1737125 | 516.2544485 | 26.17514964 | 27.01973685 | 44.82443576 |
| ENSG00000270882 | 2.912606157 | 56.47868201 | 5.85543798 | 1.047005986 | 1.000730995 | 2.861134198 |
| ENSG00000227471 | 26.21345541 | 23.61835793 | 34.15672155 | 2.094011971 | 0 | 4.768556996 |
| ENSG00000205420 | 2072.804715 | 2114.356478 | 1803.474898 | 183.2260475 | 182.133041 | 173.5754747 |
| ENSG00000186081 | 16884.37789 | 17704.52648 | 16113.18941 | 1609.2482 | 1477.078948 | 1676.62464 |
| ENSG00000186847 | 1547.564738 | 1634.801123 | 1372.1243 | 131.9227542 | 148.1081872 | 170.7143405 |
| ENSG00000270276 | 13.59216207 | 48.26360099 | 54.65075448 | 4.188023942 | 5.003654973 | 2.861134198 |
| ENSG00000128510 | 21.35911182 | 23.61835793 | 8.78315697 | 1.047005986 | 1.000730995 | 3.814845597 |
| ENSG00000186442 | 27.18432413 | 11.2957364 | 19.5181266 | 1.047005986 | 4.002923978 | 1.907422798 |
| ENSG00000187134 | 1504.846514 | 1074.121843 | 1541.932001 | 184.2730535 | 182.133041 | 137.3344415 |
| ENSG00000151632 | 15409.62831 | 12346.23989 | 14990.89713 | 2274.097001 | 1641.198831 | 1532.614219 |
| ENSG00000160712 | 29.12606157 | 50.31737125 | 25.37356458 | 4.188023942 | 5.003654973 | 4.768556996 |
| ENSG00000198074 | 246.6006546 | 202.2963701 | 298.627337 | 38.73922147 | 39.02850879 | 22.88907358 |
| ENSG00000167941 | 27.18432413 | 7.188195893 | 15.61450128 | 2.094011971 | 5.003654973 | 0 |
| ENSG00000187017 | 266.018029 | 261.8557075 | 289.84418 | 38.73922147 | 36.0263158 | 43.87072436 |
| ENSG00000166183 | 13.59216207 | 8.21508102 | 15.61450128 | 0 | 3.002192984 | 2.861134198 |
| ENSG00000241945 | 15.5338995 | 54.42491176 | 9.7590633 | 4.188023942 | 4.002923978 | 4.768556996 |
| ENSG00000224251 | 24.27171798 | 18.4839323 | 16.59040761 | 6.282035913 | 3.002192984 | 0.953711399 |
| ENSG00000270168 | 304.8527778 | 257.748167 | 283.0128357 | 50.25628731 | 42.03070177 | 56.26897255 |
| ENSG00000197915 | 52.42691083 | 58.53245227 | 47.81941017 | 10.47005986 | 10.00730995 | 7.629691194 |
| ENSG00000171903 | 47.57256723 | 31.83343895 | 34.15672155 | 8.376047884 | 8.005847956 | 5.722268395 |
| ENSG00000137801 | 1767.951937 | 1597.833258 | 1285.268637 | 312.0077837 | 272.1988305 | 318.5396073 |
| ENSG00000115844 | 74.75689136 | 56.47868201 | 53.67484815 | 9.42305387 | 21.01535089 | 7.629691194 |
| ENSG00000070915 | 262.1345541 | 225.9147281 | 325.9527142 | 52.35029928 | 68.04970763 | 51.50041556 |
| ENSG00000165949 | 572.8125442 | 503.1737125 | 460.6277877 | 76.43143695 | 148.1081872 | 102.0471197 |
| ENSG00000157168 | 283.4936659 | 271.0976737 | 235.1934255 | 60.72634716 | 52.03801172 | 57.22268395 |
| ENSG00000171658 | 280.5810598 | 227.9684983 | 252.7597395 | 57.58532921 | 55.0402047 | 51.50041556 |
| ENSG00000168481 | 23.30084926 | 10.26885128 | 16.59040761 | 1.047005986 | 2.001461989 | 7.629691194 |
| ENSG00000136235 | 132.0381458 | 82.1508102 | 96.61472667 | 20.94011971 | 35.02558481 | 11.44453679 |
| ENSG00000280441 | 23.30084926 | 44.15606048 | 9.7590633 | 6.282035913 | 3.002192984 | 7.629691194 |
| ENSG00000128564 | 12.62129335 | 14.37639179 | 13.66268862 | 1.047005986 | 6.004385967 | 1.907422798 |
| ENSG00000136244 | 37.86388004 | 48.26360099 | 40.01215953 | 10.47005986 | 16.01169591 | 1.907422798 |
| ENSG00000179431 | 285.4354034 | 277.2589844 | 239.0970508 | 79.5724549 | 48.03508774 | 53.40783836 |
| ENSG00000262681 | 33.00953645 | 28.75278357 | 26.34947091 | 5.235029928 | 8.005847956 | 6.675979795 |
| ENSG00000069812 | 205.8241684 | 179.7048973 | 192.253547 | 48.16227534 | 35.02558481 | 48.63928136 |
| ENSG00000178860 | 87.37818471 | 60.58622252 | 52.69894182 | 16.75209577 | 16.01169591 | 14.30567099 |
| ENSG00000249669 | 49.51430467 | 45.18294561 | 37.08444054 | 10.47005986 | 11.00804094 | 9.537113992 |
| ENSG00000140465 | 312.6197275 | 254.6675116 | 222.5066432 | 73.29041899 | 65.04751465 | 49.59299276 |
| ENSG00000196139 | 3044.644303 | 2544.621346 | 3105.333942 | 848.0748483 | 648.4736845 | 570.3194167 |
| ENSG00000135454 | 83.49470983 | 67.77441842 | 74.16888108 | 12.56407183 | 25.01827486 | 16.21309379 |
| ENSG00000179046 | 15.5338995 | 12.32262153 | 13.66268862 | 0 | 7.005116962 | 2.861134198 |
| ENSG00000183486 | 94.17426574 | 99.60785737 | 108.3256026 | 36.64520949 | 21.01535089 | 15.25938239 |
| ENSG00000168918 | 22.32998054 | 12.32262153 | 23.42175192 | 5.235029928 | 5.003654973 | 3.814845597 |
| ENSG00000187957 | 44.65996107 | 17.45704717 | 27.32537724 | 6.282035913 | 7.005116962 | 8.583402593 |
| ENSG00000180638 | 249.5132608 | 202.2963701 | 209.8198609 | 64.9143711 | 48.03508774 | 49.59299276 |
| ENSG00000137975 | 110.679034 | 94.47343173 | 102.4701646 | 16.75209577 | 23.01681287 | 35.28732177 |
| ENSG00000143631 | 107.7664278 | 98.58097224 | 84.90385071 | 25.12814365 | 24.01754387 | 22.88907358 |
| ENSG00000187908 | 58.25212314 | 57.50556714 | 82.95203805 | 3356.70119 | 3871.828218 | 2520.659228 |
| ENSG00000007306 | 0.970868719 | 1.026885128 | 0 | 23.03413168 | 37.0270468 | 24.79649638 |
| ENSG00000163735 | 4.854343595 | 2.053770255 | 3.90362532 | 112.0296405 | 106.0774854 | 108.7230995 |
| ENSG00000086548 | 19.41737438 | 11.2957364 | 13.66268862 | 429.2724541 | 352.2573101 | 312.8173389 |
| ENSG00000179869 | 4.854343595 | 3.080655383 | 3.90362532 | 97.37155666 | 90.06578951 | 81.06546893 |
| ENSG00000119535 | 0.970868719 | 0 | 0.97590633 | 8.376047884 | 18.0131579 | 15.25938239 |
| ENSG00000105388 | 28.15519285 | 25.67212819 | 37.08444054 | 588.4173639 | 608.4444447 | 578.9028193 |
| ENSG00000183778 | 1.941737438 | 3.080655383 | 1.95181266 | 40.83323344 | 33.02412282 | 57.22268395 |
| ENSG00000125999 | 12.62129335 | 9.241966148 | 13.66268862 | 184.2730535 | 241.1761697 | 194.5571254 |
| ENSG00000215182 | 151.4555202 | 144.790803 | 137.6027925 | 2554.694605 | 2167.583334 | 2155.387762 |
| ENSG00000162896 | 279.6101911 | 260.8288224 | 296.6755243 | 3969.199691 | 5074.706873 | 4053.273447 |
| ENSG00000064787 | 8.737818471 | 12.32262153 | 10.73496963 | 142.392814 | 199.1454679 | 154.5012467 |
| ENSG00000275212 | 17.47563694 | 3.080655383 | 5.85543798 | 118.3116764 | 114.0833334 | 145.9178441 |
| ENSG00000112303 | 0.970868719 | 2.053770255 | 5.85543798 | 29.3161676 | 39.02850879 | 41.96330157 |
| ENSG00000206531 | 1.941737438 | 0 | 3.90362532 | 24.08113767 | 26.01900586 | 17.16680519 |
| ENSG00000228113 | 1.941737438 | 2.053770255 | 1.95181266 | 29.3161676 | 23.01681287 | 13.35195959 |
| ENSG00000176533 | 0.970868719 | 1.026885128 | 1.95181266 | 12.56407183 | 13.00950293 | 13.35195959 |
| ENSG00000242770 | 2.912606157 | 3.080655383 | 8.78315697 | 41.88023942 | 45.03289475 | 57.22268395 |
| ENSG00000093134 | 3.883474876 | 1.026885128 | 0.97590633 | 12.56407183 | 14.01023392 | 29.56505338 |
| ENSG00000105851 | 0 | 1.026885128 | 3.90362532 | 20.94011971 | 10.00730995 | 15.25938239 |
| ENSG00000118322 | 6.796081033 | 16.43016204 | 14.63859495 | 107.8416165 | 126.0921053 | 103.0008311 |
| ENSG00000047457 | 9.70868719 | 8.21508102 | 5.85543798 | 63.86736512 | 89.06505851 | 59.13010675 |
| ENSG00000163734 | 51.45604211 | 43.12917536 | 42.93987852 | 415.6613763 | 385.2814329 | 415.8181701 |
| ENSG00000003137 | 75.72776008 | 50.31737125 | 65.38572411 | 539.2080826 | 553.40424 | 563.6434369 |
| ENSG00000124664 | 2.912606157 | 1.026885128 | 11.71087596 | 46.06826336 | 44.03216376 | 40.05587877 |
| ENSG00000126583 | 0 | 3.080655383 | 1.95181266 | 8.376047884 | 16.01169591 | 15.25938239 |
| ENSG00000205670 | 0.970868719 | 3.080655383 | 4.87953165 | 48.16227534 | 18.0131579 | 2.861134198 |
| ENSG00000198788 | 1.941737438 | 8.21508102 | 0 | 18.84610774 | 25.01827486 | 31.47247617 |
| ENSG00000184454 | 2.912606157 | 2.053770255 | 0 | 8.376047884 | 16.01169591 | 12.39824819 |
| ENSG00000275395 | 0 | 5.134425638 | 2.92771899 | 20.94011971 | 23.01681287 | 15.25938239 |
| ENSG00000130433 | 4.854343595 | 3.080655383 | 4.87953165 | 28.26916161 | 37.0270468 | 29.56505338 |
| ENSG00000232316 | 9.70868719 | 8.21508102 | 4.87953165 | 54.44431125 | 57.04166669 | 55.31526115 |
| ENSG00000154274 | 8.737818471 | 2.053770255 | 1.95181266 | 35.59820351 | 29.02119884 | 27.65763058 |
| ENSG00000197353 | 769.8988942 | 713.6851636 | 986.6412996 | 5273.769149 | 6365.649856 | 5216.801354 |
| ENSG00000233539 | 7.766949752 | 6.161310765 | 8.78315697 | 55.49131723 | 41.02997078 | 58.17639535 |
| ENSG00000137745 | 53.39777955 | 60.58622252 | 40.98806586 | 320.3838316 | 363.265351 | 347.1509493 |
| ENSG00000143416 | 0.970868719 | 1.026885128 | 5.85543798 | 11.51706584 | 24.01754387 | 16.21309379 |
| ENSG00000196482 | 3.883474876 | 2.053770255 | 0 | 11.51706584 | 12.00877193 | 13.35195959 |
| ENSG00000106258 | 28.15519285 | 22.59147281 | 42.93987852 | 197.8841313 | 227.1659358 | 147.8252669 |
| ENSG00000256574 | 2.912606157 | 3.080655383 | 0 | 11.51706584 | 19.0138889 | 5.722268395 |
| ENSG00000160870 | 0.970868719 | 2.053770255 | 3.90362532 | 15.70508978 | 19.0138889 | 6.675979795 |
| ENSG00000204385 | 37.86388004 | 45.18294561 | 59.53028613 | 232.4353288 | 340.2485381 | 269.900326 |
| ENSG00000019186 | 12.62129335 | 5.134425638 | 15.61450128 | 68.05538906 | 64.04678365 | 61.99124095 |
| ENSG00000000971 | 4.854343595 | 3.080655383 | 14.63859495 | 35.59820351 | 51.03728072 | 41.96330157 |
| ENSG00000268104 | 400.9687809 | 424.1035577 | 370.8444054 | 2178.819456 | 2385.742691 | 2148.711782 |
| ENSG00000002726 | 127.1838022 | 174.5704717 | 138.5786989 | 738.1392198 | 990.7236846 | 694.3018986 |
| ENSG00000135074 | 1.941737438 | 6.161310765 | 5.85543798 | 18.84610774 | 29.02119884 | 28.61134198 |
| ENSG00000102359 | 88.34905343 | 85.23146558 | 94.66291401 | 486.8577833 | 497.3633043 | 485.4391022 |
| ENSG00000159239 | 4.854343595 | 5.134425638 | 2.92771899 | 12.56407183 | 27.01973685 | 28.61134198 |
| ENSG00000230606 | 1.941737438 | 3.080655383 | 4.87953165 | 20.94011971 | 19.0138889 | 12.39824819 |
| ENSG00000283992 | 0.970868719 | 6.161310765 | 7.80725064 | 33.50419154 | 26.01900586 | 19.07422798 |
| ENSG00000154556 | 2.912606157 | 12.32262153 | 6.83134431 | 39.78622745 | 39.02850879 | 36.24103317 |
| ENSG00000235836 | 1.941737438 | 5.134425638 | 1.95181266 | 14.6580838 | 14.01023392 | 18.12051659 |
| ENSG00000164120 | 78.64036624 | 49.29048612 | 77.09660007 | 350.7470052 | 401.2931288 | 321.4007415 |
| ENSG00000259078 | 0 | 3.080655383 | 3.90362532 | 5.235029928 | 17.01242691 | 13.35195959 |
| ENSG00000277701 | 5.825212314 | 4.10754051 | 7.80725064 | 35.59820351 | 31.02266083 | 22.88907358 |
| ENSG00000204616 | 963.1017693 | 1039.207749 | 1402.377396 | 5898.831723 | 5750.200295 | 5402.775077 |
| ENSG00000115525 | 4.854343595 | 3.080655383 | 1.95181266 | 11.51706584 | 19.0138889 | 18.12051659 |
| ENSG00000006047 | 2.912606157 | 4.10754051 | 4.87953165 | 26.17514964 | 21.01535089 | 11.44453679 |
| ENSG00000253196 | 3.883474876 | 10.26885128 | 10.73496963 | 36.64520949 | 41.02997078 | 40.05587877 |
| ENSG00000079257 | 1851.446647 | 1975.726985 | 2201.64468 | 9738.202672 | 9628.032899 | 9156.583144 |
| ENSG00000231226 | 27.18432413 | 52.3711415 | 40.01215953 | 197.8841313 | 177.129386 | 180.2514545 |
| ENSG00000137868 | 806.7919055 | 830.7500682 | 1075.448776 | 4135.673643 | 4413.223686 | 4044.690044 |
| ENSG00000104368 | 863.1022912 | 886.201865 | 955.412297 | 3740.952386 | 4595.356727 | 4157.227989 |
| ENSG00000240996 | 0.970868719 | 4.10754051 | 2.92771899 | 15.70508978 | 10.00730995 | 10.49082539 |
| ENSG00000232931 | 390.289225 | 498.0392868 | 498.6881346 | 1994.546402 | 2103.536551 | 2177.323124 |
| ENSG00000134827 | 3.883474876 | 6.161310765 | 7.80725064 | 30.36317358 | 26.01900586 | 23.84278498 |
| ENSG00000140835 | 4.854343595 | 4.10754051 | 2.92771899 | 23.03413168 | 20.01461989 | 10.49082539 |
| ENSG00000198535 | 10.67955591 | 9.241966148 | 10.73496963 | 37.69221548 | 58.04239768 | 41.96330157 |
| ENSG00000265972 | 404.8522558 | 380.9743823 | 346.4467471 | 1538.051793 | 1916.399855 | 1627.031647 |
| ENSG00000174501 | 368.9301132 | 417.9422469 | 433.3024105 | 1822.837421 | 1815.326024 | 1740.523304 |
| ENSG00000162366 | 143.6885704 | 210.5114511 | 166.8799824 | 636.5796392 | 857.6264623 | 798.2564411 |
| ENSG00000064692 | 5.825212314 | 0 | 2.92771899 | 12.56407183 | 12.00877193 | 13.35195959 |
| ENSG00000116833 | 28.15519285 | 30.80655383 | 45.86759751 | 150.7688619 | 147.1074562 | 144.0104213 |
| ENSG00000080031 | 55.33951698 | 57.50556714 | 70.26525576 | 226.1532929 | 252.1842106 | 286.1134198 |
| ENSG00000005249 | 6.796081033 | 2.053770255 | 8.78315697 | 30.36317358 | 26.01900586 | 17.16680519 |
| ENSG00000127324 | 18.44650566 | 11.2957364 | 14.63859495 | 62.82035913 | 53.03874271 | 66.75979795 |
| ENSG00000169031 | 29.12606157 | 32.86032408 | 43.91578485 | 131.9227542 | 188.137427 | 108.7230995 |
| ENSG00000145287 | 17.47563694 | 7.188195893 | 17.56631394 | 62.82035913 | 58.04239768 | 49.59299276 |
| ENSG00000179362 | 9.70868719 | 8.21508102 | 11.71087596 | 48.16227534 | 27.01973685 | 43.87072436 |


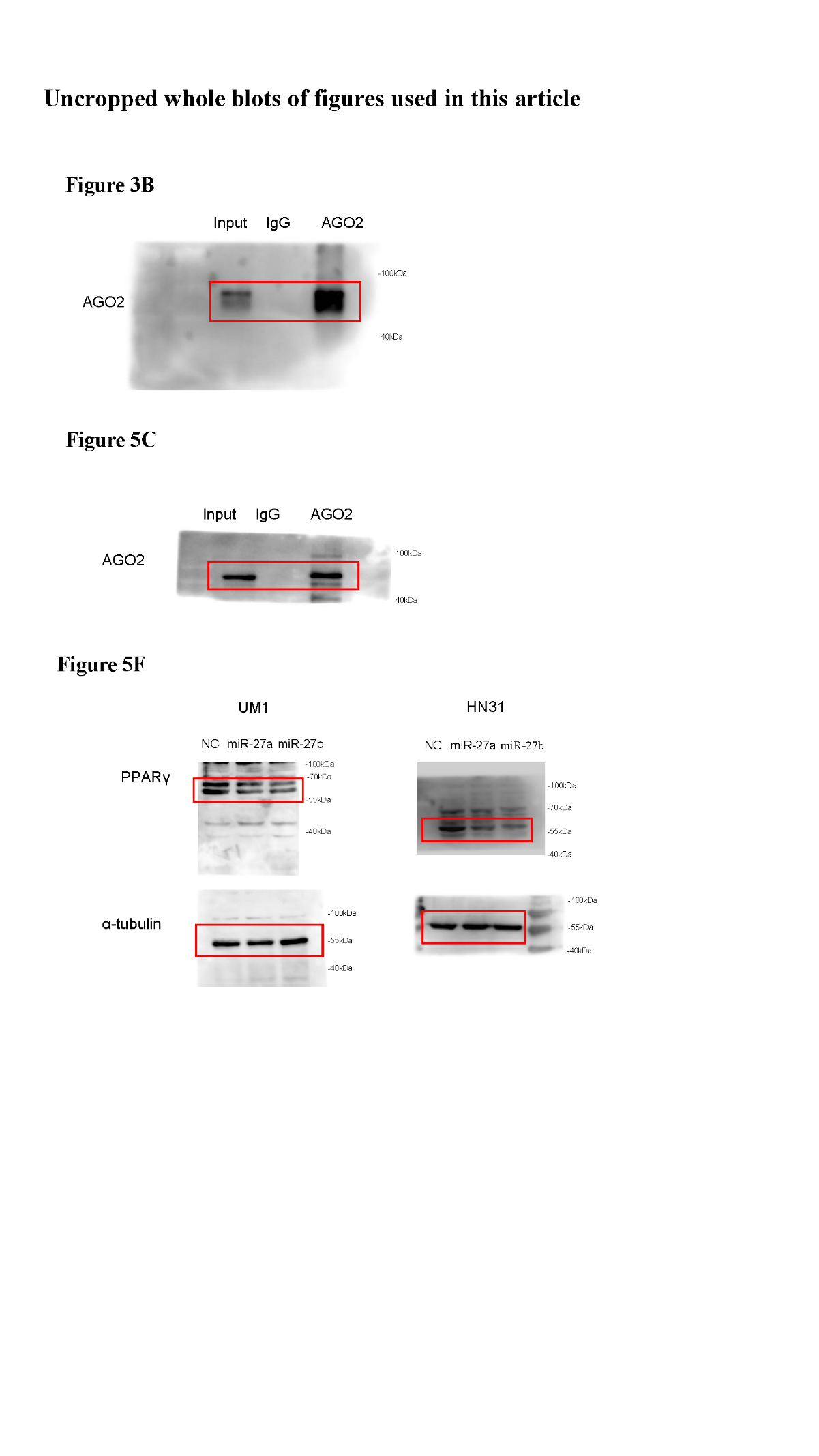


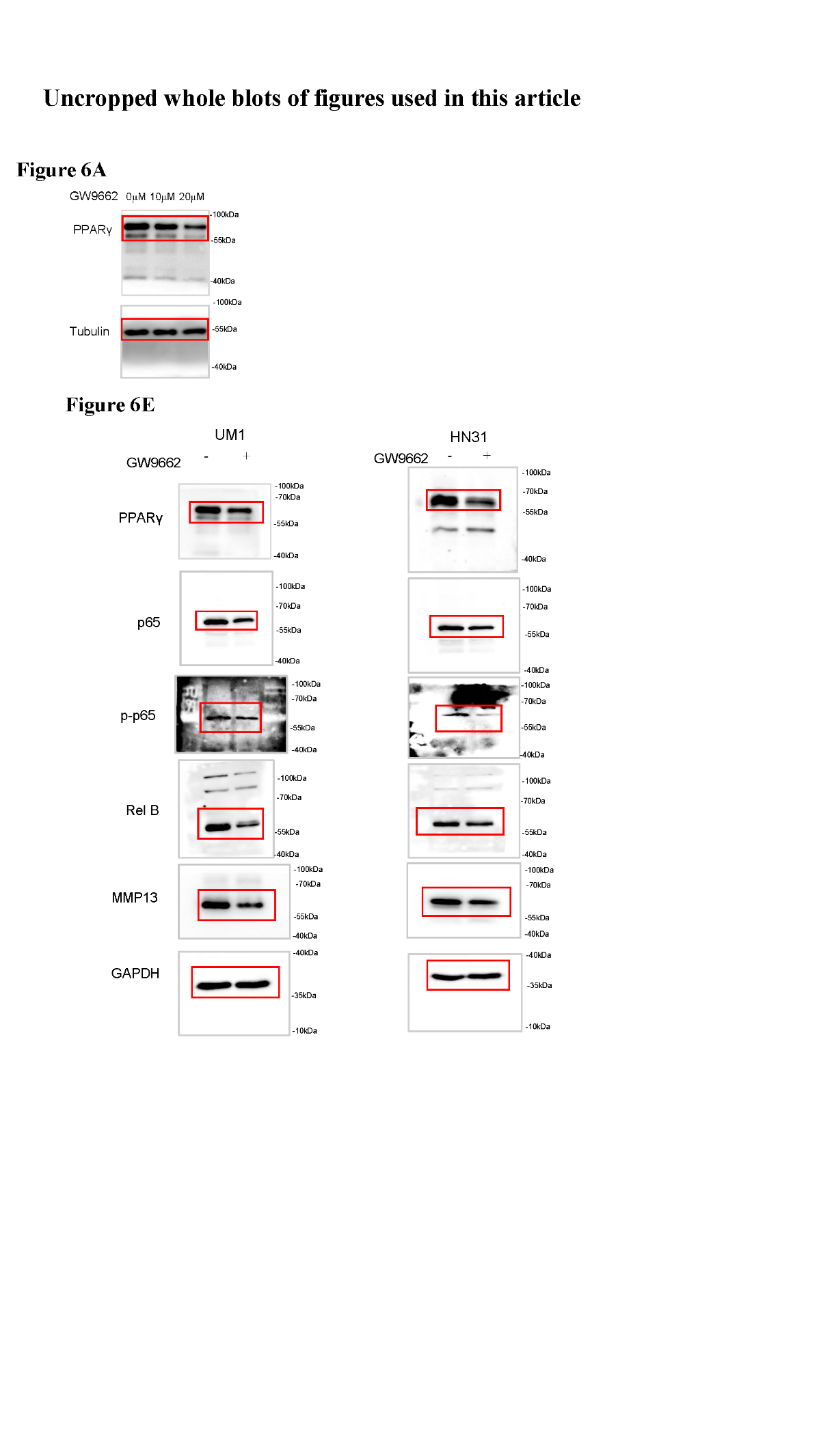


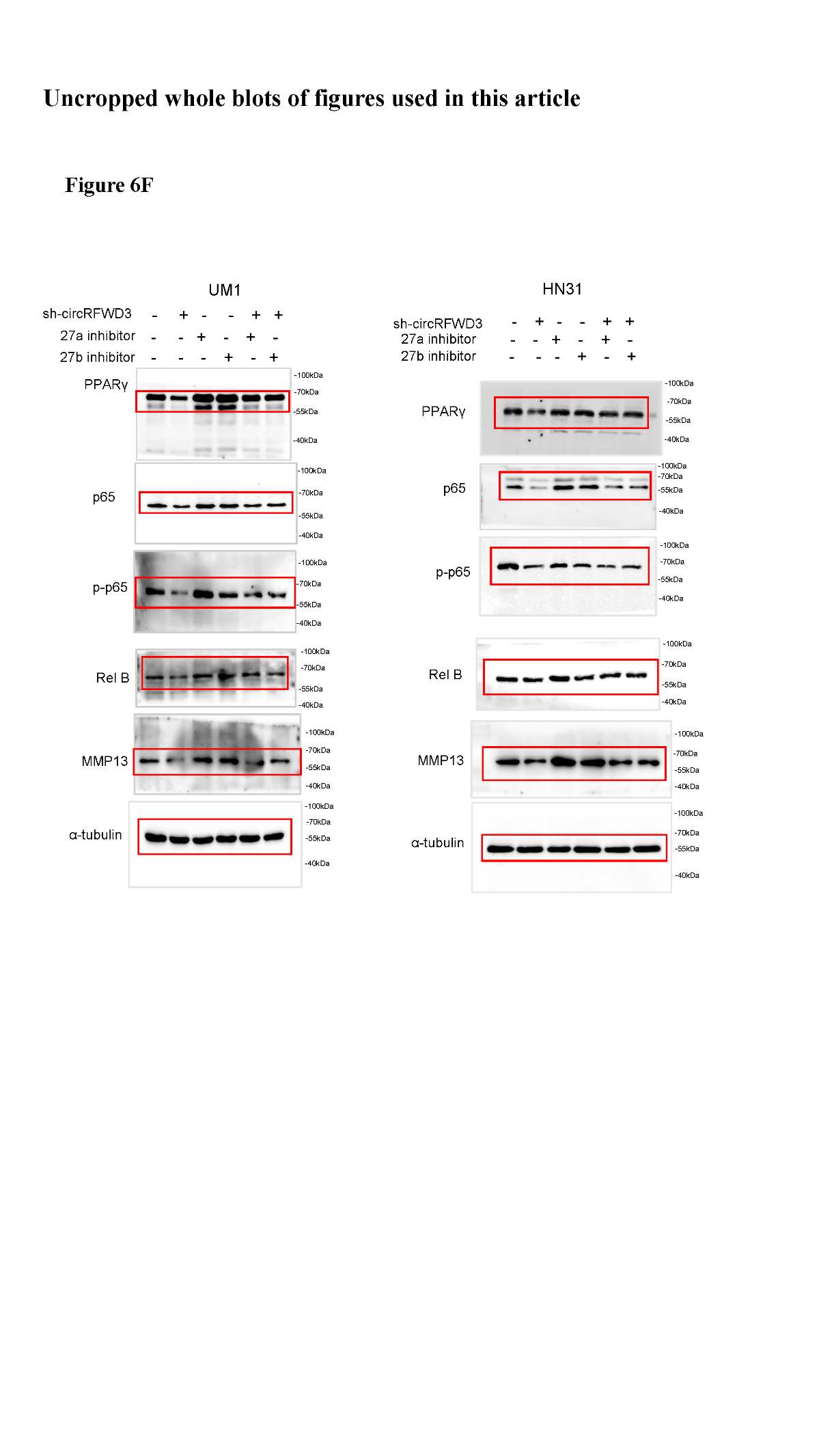


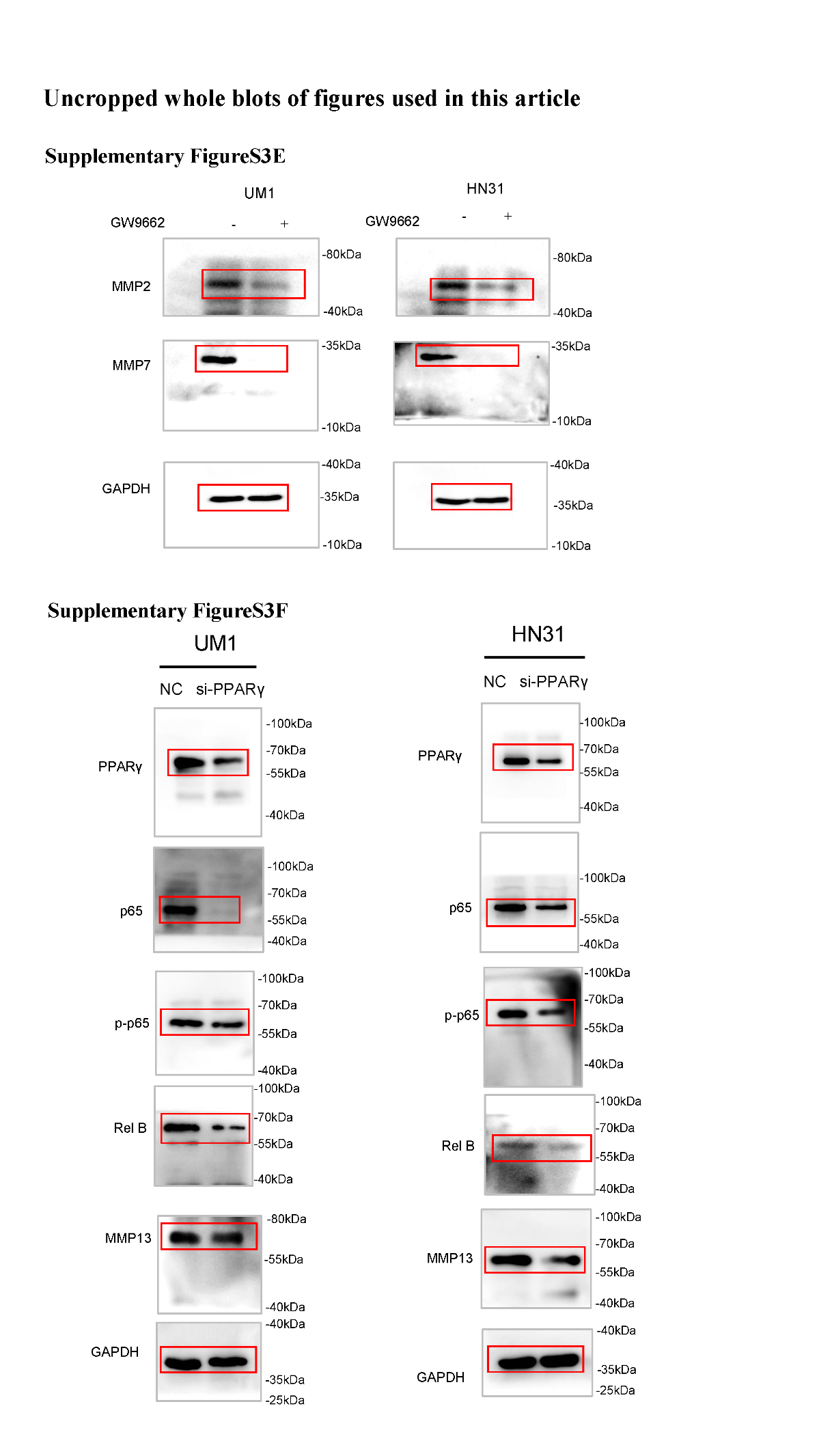

Supplement: Supplementary file 1 — Appendix file of circRFWD3 with original western blots [file 41420_2022_1066_MOESM1_ESM.docx]
